# Supplementary material for: Massively targeted evaluation of therapeutic CRISPR off-targets in cells
Source: Nat Commun. 2022 Jul 13;13:4049. doi: 10.1038/s41467-022-31543-6 (PMC9279339; doi:10.1038/s41467-022-31543-6)
Supplement: Supplementary file 1 — Supplementary Information [file 41467_2022_31543_MOESM1_ESM.pdf]

SUPPLEMENTARY INFORMATIONS FOR

MASSIVELY TARGETED EVALUATION OF THERAPEUTIC CRISPR OFF-TARGETS IN CELLS

Xiaoguang Pan[1,3,*], Kunli Qu[1,2,3,*], Hao Yuan[4,1,*], Xi Xiang[1,2,*], Christian Anthon[5,*], Liubov Pashkova[5], Xue Liang[1,3], Peng Han[1,3], Giulia I. Corsi[5], Fengping Xu[1,4,6], Ping Liu[6,7], Jiayan Zhong[6,7], Yan Zhou[2], Tao Ma[6,7], Hui Jiang[6,7], Junnian Liu[1], Jian Wang[6], Niels Jessen[2,8], Lars Bolund[1,2], Huanming Yang[6,9], Xun Xu[6,10], George M. Church[11,#], Jan Gorodkin[5,#], Lin Lin[2,8,#], Yonglun Luo[1,2,4,6,8,9#]

[1]Lars Bolund Institute of Regenerative Medicine, Qingdao-Europe Advanced Institute for Life Sciences, BGI-Qingdao, BGI-Shenzhen, Qingdao, China

[2]Department of Biomedicine, Aarhus University, Aarhus, Denmark

[3]Department of Biology, Copenhagen University, Copenhagen, Denmark

[4]College of Life Sciences, University of Chinese Academy of Sciences, Beijing, China.

[5]Center for non-coding RNA in Technology and Health, Department of Veterinary and Animal Sciences, Faculty of Health and Medical Sciences, University of Copenhagen, Frederiksberg, Denmark.

[6]BGI-Research, BGI-Shenzhen, Shenzhen, China

[7]MGI, BGI-Shenzhen, Shenzhen, China

[8]Steno Diabetes Center Aarhus, Aarhus University, Aarhus, Denmark

[9]IBMC-BGI Center, the Cancer Hospital of the University of Chinese Academy of Sciences (Zhejiang Cancer Hospital), Institute of Basic Medicine and Cancer (IBMC), Chinese Academy of Sciences, Hangzhou, Zhejiang 310022, China.

[10]Guangdong Provincial Key Laboratory of Genome Read and Write , BGI-Shenzhen, Shenzhen, China

[11]Department of Genetics, Blavatnik Institute, Harvard Medical School, Boston, MA, USA

* These authors contributed equally: Xiaoguang Pan, Kunli Qu, Hao Yuan, Xi Xiang, Christian Anthon

# All correspondence should be addressed to George M. Church [gc@hms.harvard.edu], Jan Gorodkin [gorodkin@rth.dk], Lin Lin [lin.lin@biomed.au.dk], and Yonglun Luo (lead contact) [alun@biomed.au.dk].

This supplementary document contains:

Part 1. Supplementary Notes 1-2

Part 2. Supplementary Figures and Legends

Part 3. Supplementary References

PART 1. SUPPLEMENTARY NOTES

SUPPLEMENTARY NOTE 1: A DETAIL OVERVIEW OF THE SURRO-SEQ EXPERIMENTAL PROCEDURE

SURRO-seq contains nine major steps (**see Fig.1**). During the revision of this article, we propose another two-step cloning strategy for generation of the SURRO-seq library to overcome the limitation of oligonucleotide length (**Supplementary Figure S17**).

Step 1: Selection of potential off-target sites to be evaluated by SURRO-seq.
To select potential OTs to be evaluated and validated by SURRO-seq, these pOTs could be those identified by unbiased genome-scale screening approaches (summarized in **Table S1**) or predicted with computational tools, just to mention a few: integrated prediction sites by CRISPOR [1], CRISPRoff [2], Cas-OFFinder [3]. There is no limitation of which sequences to be inserted into the surrogate position in the SURRO-seq system.

Step 2: Design and synthesis SURRO-seq oligonucleotides.
Each SURRO-seq oligonucleotide is 170 nt, containing two primer binding sites (PBS), two BsmbI cloning sites, RGN gRNA spacer (20 nt), gRNA scaffold sequences, 10 nt barcode and 27 nt surrogate off-target site. Oligonucleotides longer than 170 nt will increase both cost and synthesis errors. The 10-nt barcode preceding the surrogate protospacer in the surrogate vector (**Fig.1**) is for overcoming the alignment problem with indel reads (**Fig. S2**). Theoretically a 10-nt barcode will allow us to measure half million (4^{10}) OTs simultaneously. The SURRO-seq oligo nucleotides can be synthesized individually or using array-based oligo pool synthesis. Vector cloning is based on BsmBI-mediated Golden-Gate Assembly. It is important to avoid sites containing a BsmBI restriction enzyme recognition site.

Step 3: Clone SURRO-seq oligonucleotides into the lentiviral vector [Addgene 170459] by golden gate assembly
The synthetic SURRO-seq oligonucleotides are amplified by PCR using the same protocol described previously[4]. A detail protocol is also shared in protocols.io (**dx.doi.org/10.17504/protocols.io.bt9jnr4n**). The synthetic SURRO-seq DNA is inserted downstream of a human U6 promoter of a lentiviral vector, which we have generated previously and shared in Addgene (plasmid ID: 170459). In addition to Golden Gate Assembly, this lentiviral vector expresses two protein markers: an enhanced green

fluorescent protein for quantification of viral titer by flow cytometry and a puromycin selection marker for enrichment of RGN-edited cells.

Step 4: Lentiviral packaging

The SURRO-seq plasmid DNA pool is packaged into lentivirus using a standard lentivirus packaging protocol. It is not necessary to concentrate the lentivirus particles. We filtered the crude virus with a 0.45 um syringe filter and save crude virus at -80 until use. It is however essential to quantify titer based on quantification of GFP+ cells.

Step 5: SURRO-seq lentivirus library transduction

For lentivirus transduction, it is important that transduction is carried out at low multiplicity of infection (MOI) to maximize the number of cells with a single integration. Second, transduction should be performed with high coverage of the library. In our study, we have formed the experiment with a 4000-fold coverage. We recommend that at least 500-fold coverage of the library should be used. In this study, we have used a conventional HEK293T cells expressing SpCas9. One limitation of using SpCas9-expressing cells is that the lentivirus genomics DNA could be edited before integrating into the genome. Alternatively, the SURRO-seq lentivirus library can be integrated into a wild type cells, followed by expression of the Cas9 protein.

Step 6: Enrichment of SURRO-seq transduced cells

Two days after transduction, cells were switched to selection growth medium (+puromycin) and cultured with selective medium for 6 addition days. Cells were passaged upon 80% confluence. 8 days after transduction, only cells with the SURRO-seq lentiviral vector stably integrated in the genome are remained in culture.

Step 7: Amplification of surrogate sites from transduced cells by PCR

Genomic DNA is purified from post-selection transduced cells. The surrogate sites are amplified from the genomic DNA using a pair of primers which amplify the gRNA expression cassette and the surrogate off-target sites. The PCR condition has to be optimized for user' specific DNA polymerase. The following conditions should be met to ensure that PCR is amplified at least 500-fold coverage of cells:

[Genome (G) = 3pg/cell]

[e.g Library size (K) = 1000 vectors]

[Cell coverage (C) = 500 cells]

[Total genomic DNA for PCR (M) = K*G*C=1000*3*500 =1.5 ug]

Step 8: Targeted deep sequencing

For targeted deep sequencing, at least pair end 150 cycles (PE150) should be used. The sequencing coverage should be at least 500-fold of the library.

Step 9: Data analysis

As individual SURRO-seq vector carries a unique 10-nt barcode, after data quality control, the merged pair-end reads were separated based on barcodes. Reads were then aligned to individual SURRO-seq reference sequences to identify WT and indel reads. Computing codes for the indel data analysis are shared in GitHub.

Supplementary Note 2: A statistical model for defining pOTs with significant indels

The SURRO-seq method directly measures RGN-induced indels in cells and compares to unedited MOCK cells. However, three steps could introduce indels independent of RGNs.

First, the synthesis of oligo pool is a step that can introduce indels. Indels generated during oligo synthesis can be easily removed, as these type of indels will appear in both RGN-edited cells and MOCK cells. See method for the removal of synthesized indels. Second, PCR introduced indels. To avoid PCR-introduced indels, it is essential to use proof-reading DNA polymerase. Despite that, very low random indels could still be introduced by PCRs. Third, sequencing introduced indels. The indel rate per 100 sequences bases for high throughput sequencing machine is approximately 0.02[5].

Based on these, it is essential that certain criteria are introduced to judge whether the indels observed for an OT are statistically significant. For each surrogate off-targets site, the data structure falls into a 2×2 contingency table model. For RGN-edited and MOCK cells, total reads are categorized into indel reads or wildtype (WT) reads. We thus perform Fisher's Exact Test between RGN and MOCK to obtained Fisher test p values. Next, to decrease false positive rate, we run the Benjamini-Hochberg (BH) procedure based on the Fisher exact test p values. Third, we calculate fold change (FC) of indel frequencies (IF%) between RGN and MOCK. An OT with FC (RGN vs. MOCK, IF%) > 2 and BH adj. p value < 0.05 is considered statistically significant. We validate that the SURRO-seq is highly accurate

(nearly 100% concordance with targeted in-cell RGN OT detection method, T7E1) and has the lowest false positive rate as compared to GUIDE-seq and CIRCLE-seq.

PART 2. SUPPLEMENTARY FIGURES AND LEGENDS

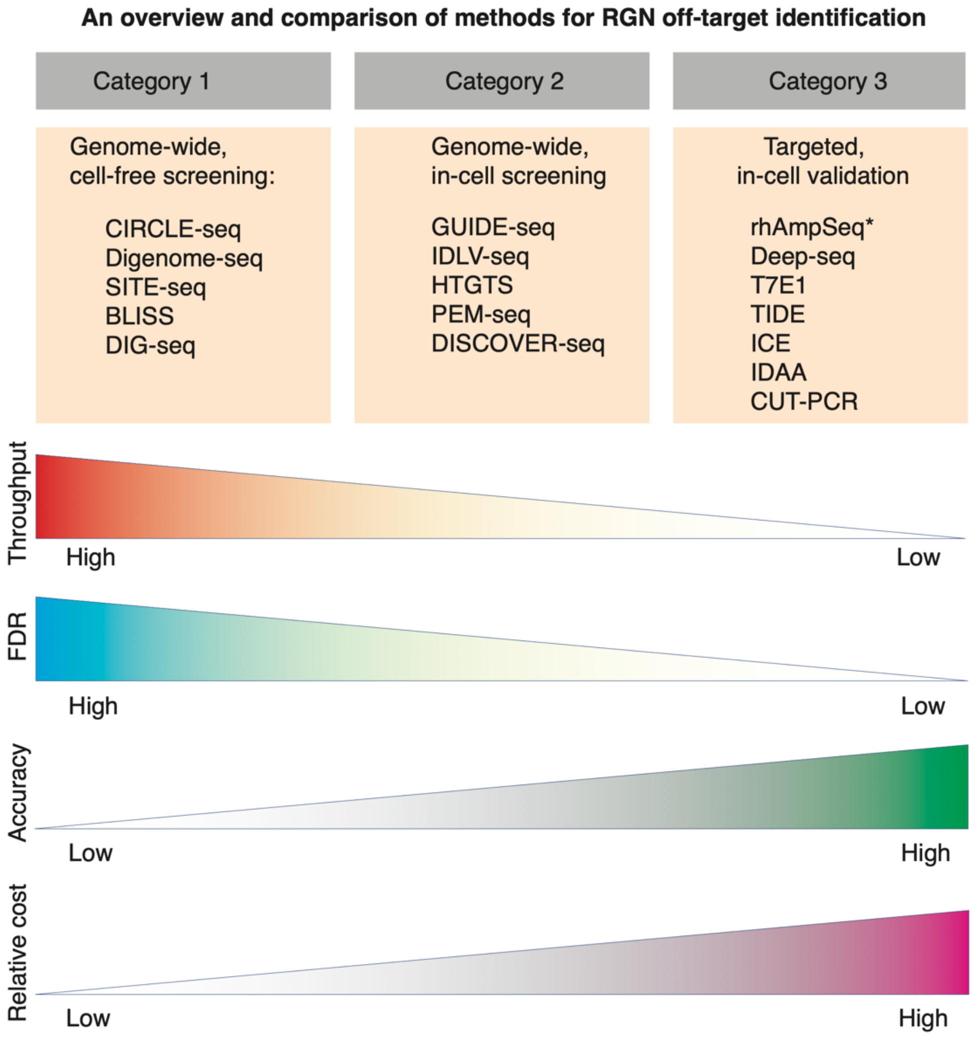

Supplementary Figure S1. Overview and comparison of RGN Off-target screening and evaluation method according to their throughput, false discovery rate (FDR), accuracy and relative cost. Detailed list and referenced of the methods mentioned here are listed in Supplementary Data1. The throughput is estimated by the number of RGNs can be performed per study or experiment. FDR is estimated based on the assumption that DNA of cell-free screening methods is more accessible to the RGNs. COST is estimated as cost for evaluating one off-target. *, rhAmpSeq is a significantly improved and scalable targeted amplicon sequencing method.

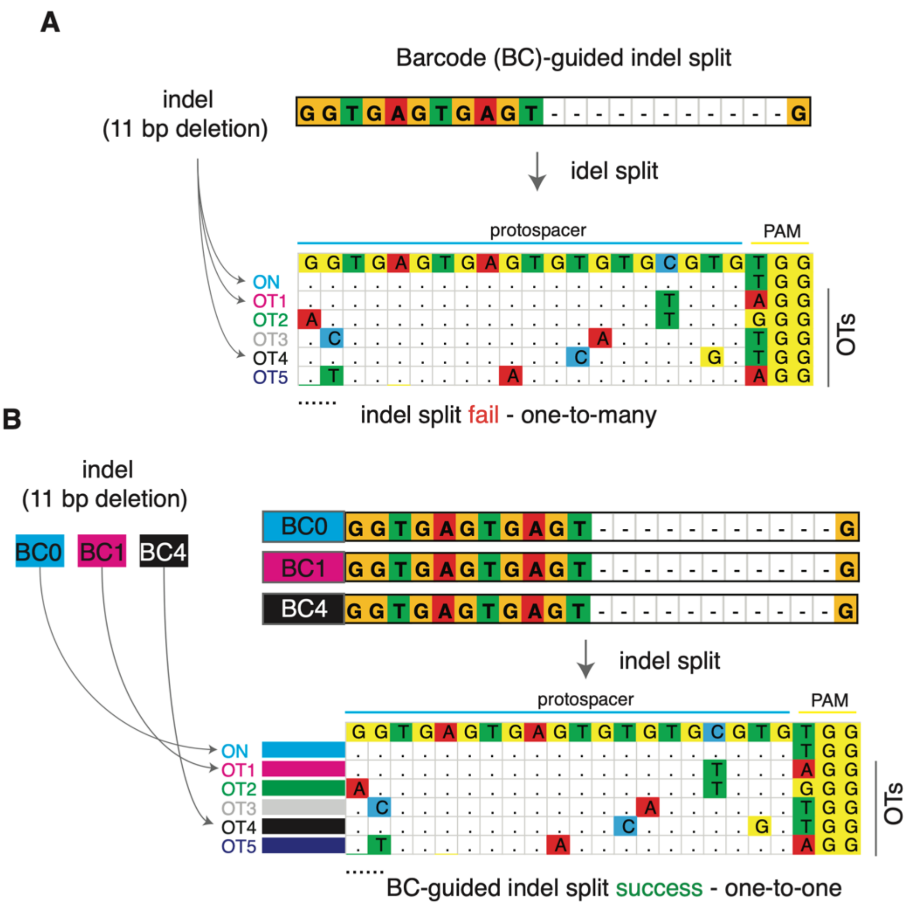

Supplementary Figure S2. Schematic illustration of enabling indel splitting by the inclusion of barcodes.

A. Example of indel collision without barcoding of surrogate sites. Indel reads of a 11-bp deletion could be mapped to ON target, OT1 and OT4.

B. Example of precise indel splitting through the introduction of barcodes. In this case, indel reads of a 11-bp deletion will carry a specific barcode. This enables the precise splitting of all indels to corresponding surrogate sites.

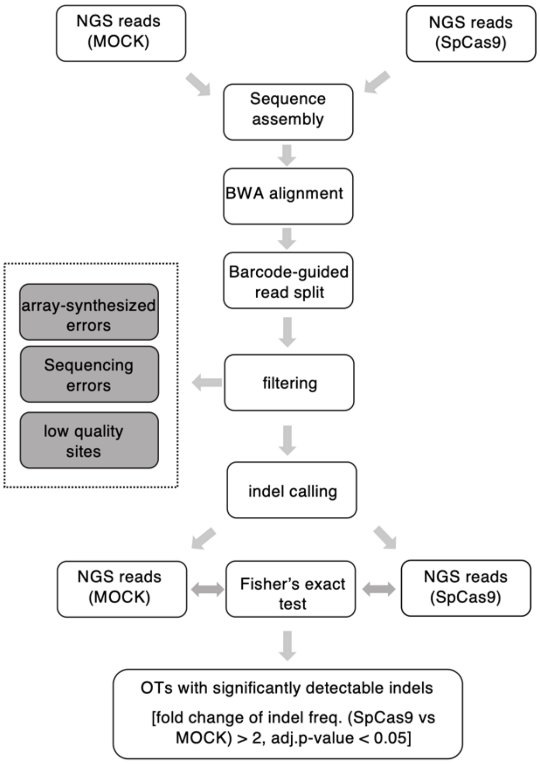

Supplementary Figure S3. A flowchart of SURRO-seq data processing and analysis

MOCK, wildtype cells transduced with the SURRO-seq library. SpCas9, HEK293T cells expressing the SpCas9 protein. OTs, off-targets. Gray boxes are three steps for filtering the RGN-independent indels caused by synthesis, sequencing and low-quality sites.

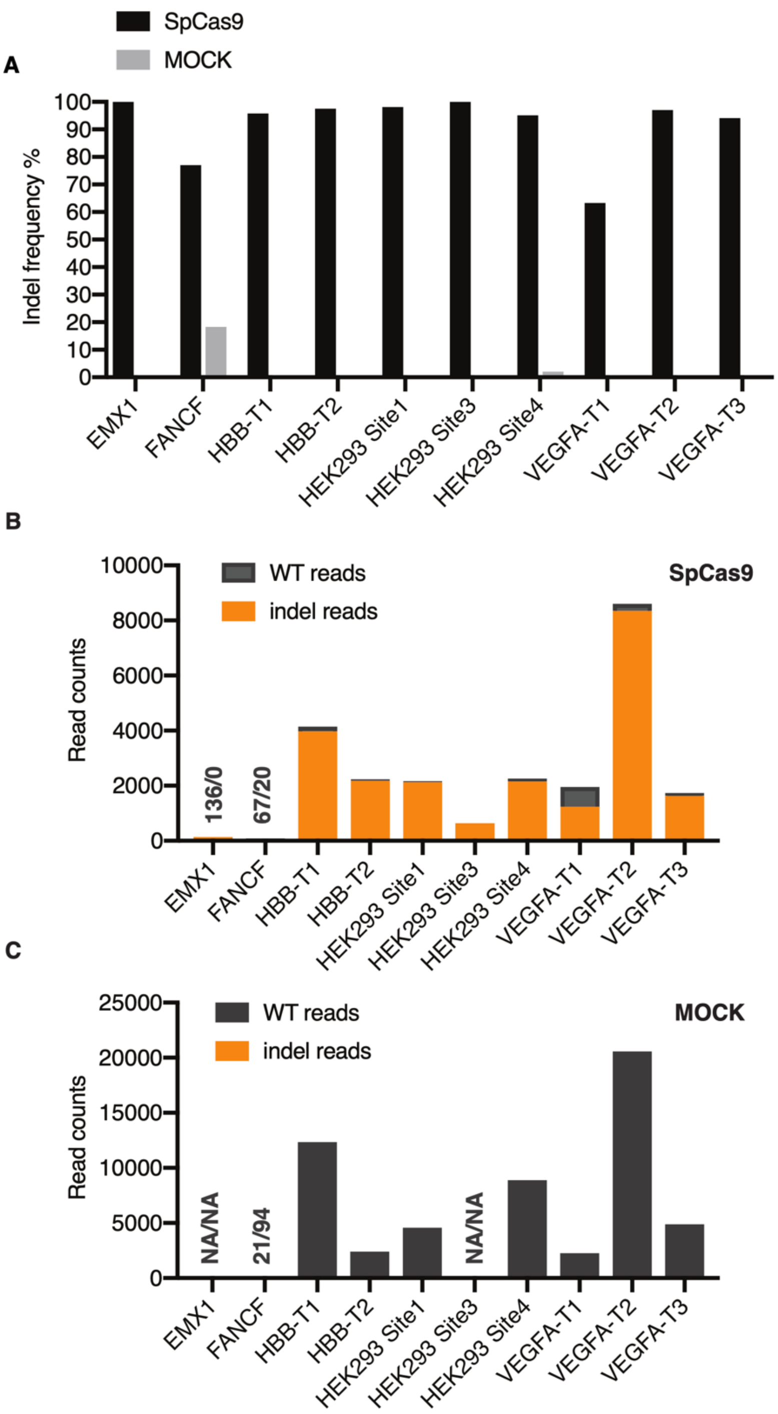

Supplementary Figure S4. Deep sequencing analysis of Library A (LibA) data

A. On-target efficiency of the 11 RGN gRNAs in HEK293T-SpCas9 cells and wildtype cells (MOCK). Indel frequency is % indel reads of total reads.

B. Bar plot of indel reads and total ready for the 11 on-target RGNs in HEK293T-SpCas9 cells.

C. Bar plot of indel reads and total ready for the 11 on-target RGNs in WT cells. Numbers are indel (lower) and WT (upper) reads for EMX1 and FANCF. NA, value not available.

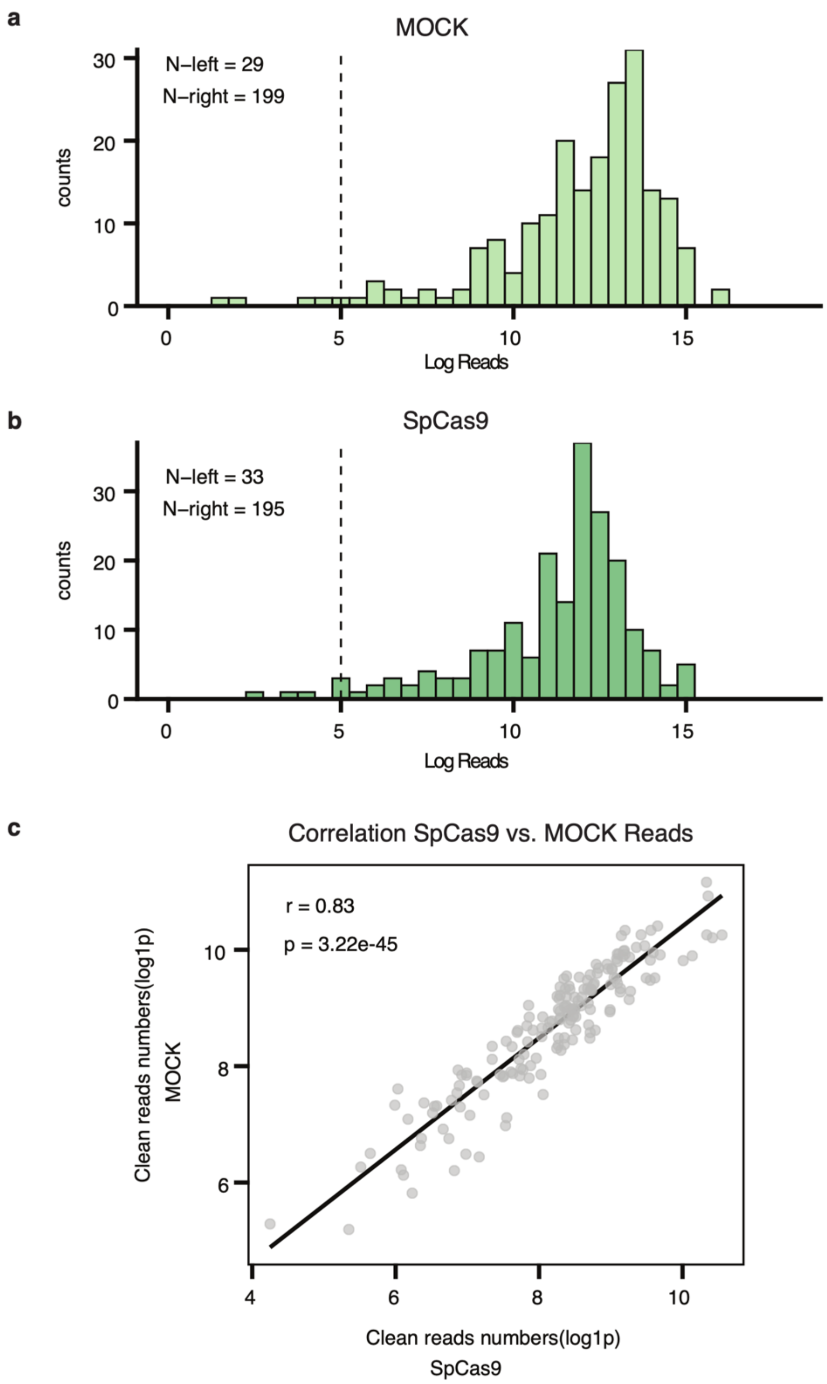

Supplementary Figure S5. QC of deep sequencing data of LibA.

A. Histogram of Log Reads of all surrogate sites included in LibA in MOCK. Dash line is cut off of reads = 32.

B. Histogram of Log Reads of all surrogate sites included in LibA in SpCas9 cells. Dash line is cut off of reads = 32.

C. Dot plot of Log1P (clean reads+1) reads in MOCK and SpCas9. The r value is Pearson's coefficient, with p value tested with t-distribution.

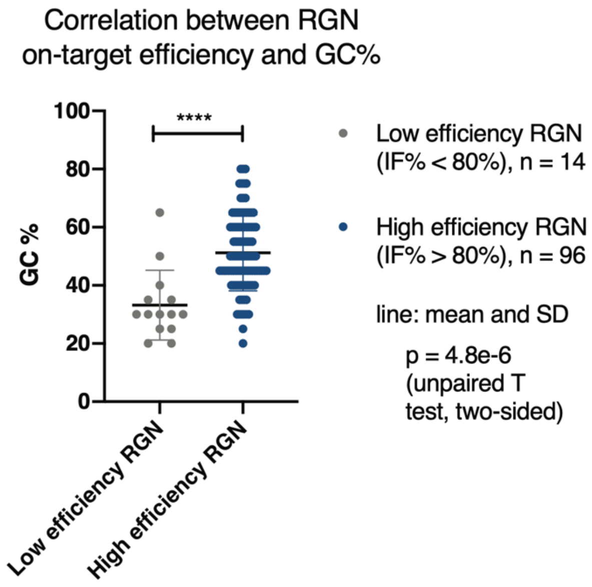

Supplementary Figure S6. Correction between RGN on-target efficiency and GC content. All 110 RGNs in LibB are grouped into high and low efficiency based on a cutoff of indel frequency of 80%. Values are presented as mean and 1 SD. P value is derived from two-sided, unpaired T test.

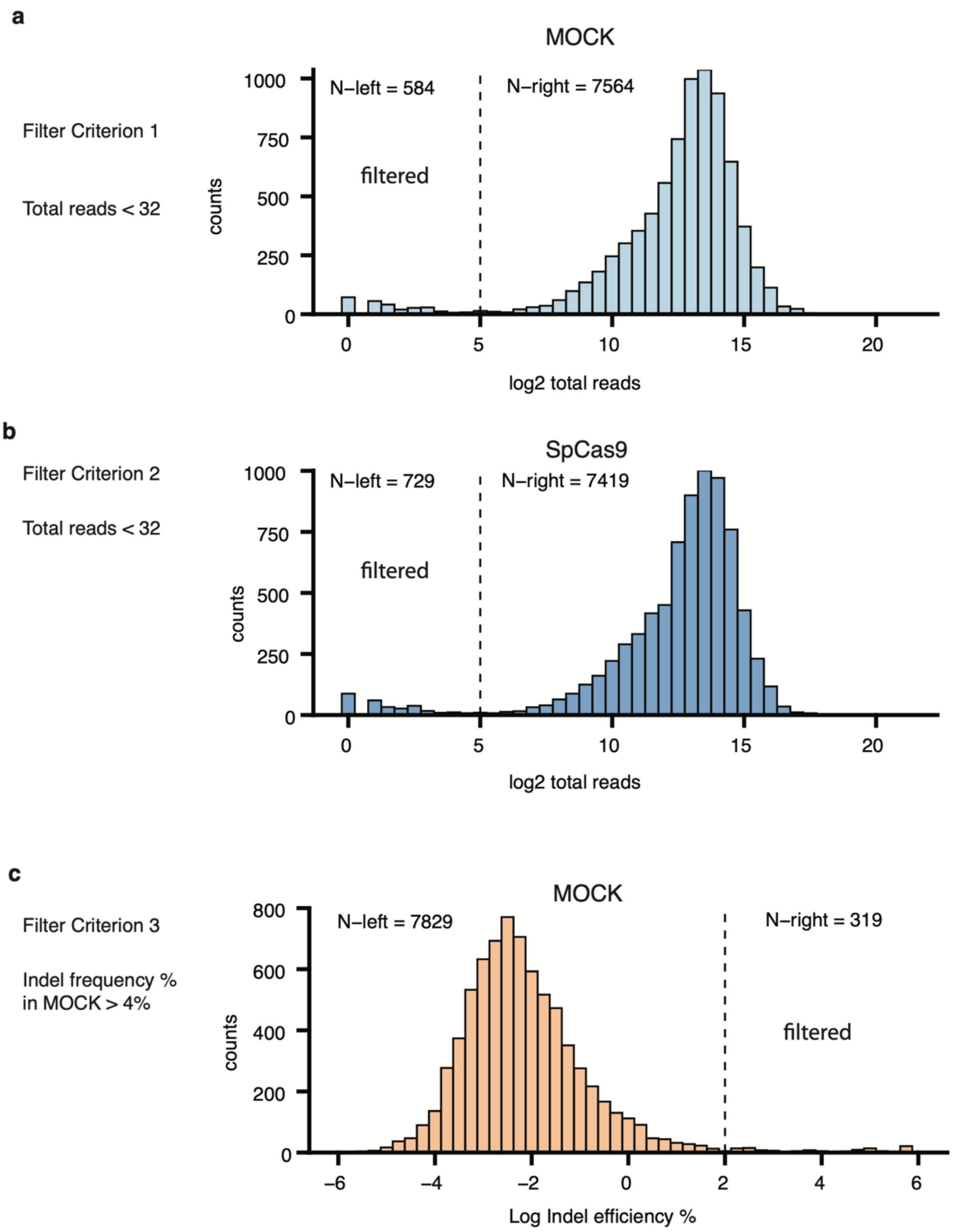

Supplementary Figure S7. QC of deep sequencing data of LibB

A. Histogram of Log Reads of all surrogate sites included in LibA in MOCK. Dash line is cut off of reads = 32.

B. Histogram of Log Reads of all surrogate sites included in LibA in SpCas9 cells. Dash line is cut off of reads = 32.

C. Dot plot of log indel frequency % in MOCK, cutoff = 4%.

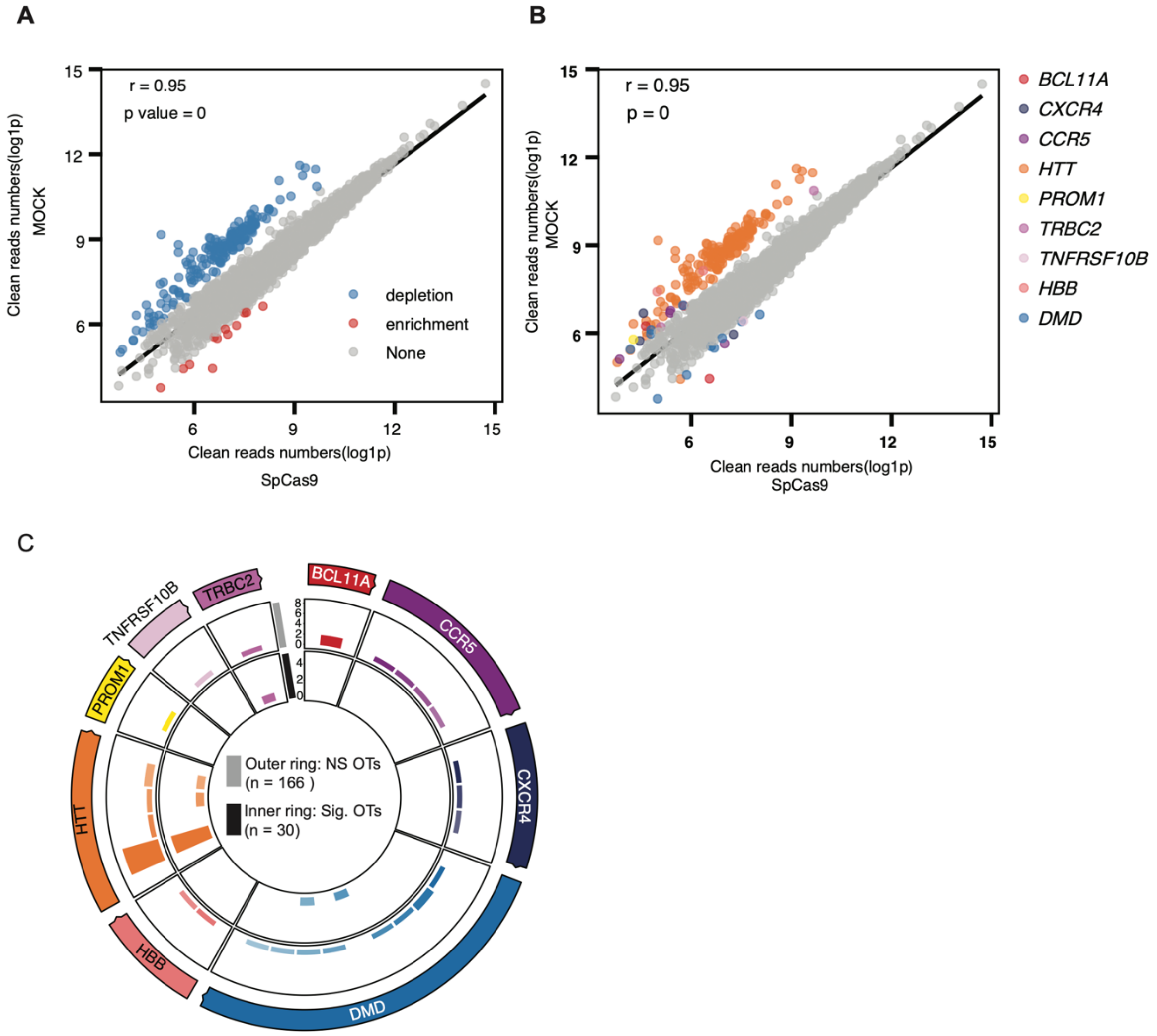

Supplementary Figure S8. Scatter plot of log1P in MOCK and SpCas9. A. Surrogate sites with over 2-fold changes in reads (log 2) between MOCK and SpCas9 were filtered. Enrichment: fold changes (SpCas9 vs. MOCK) >= 2; Depletion: fold changes (MOCK vs. SpCas9) >= 2. Data are presented as Pearson's coefficient r value (t-distribution test). B. Scatter plot of the log 2 reads of surrogate sites in SpCas9 and MOCK cells. Depletion and enrichment sites colored by the RGN on-target genes. Data are presented as Pearson's coefficient r value (t-distribution test). C. Circo plot of the number of RGN OTs with significantly (adj. $P < 0.05$, Fold change of indel frequency (IF%) in SpCas9 vs. MOCK > 2; inner circle) and non-significantly (outer circle) detectable indels. P values for comparison between IF% SpCas9 and IF% MOCK are derived from Benjamini and Hochberg (BH)-adjusted Fishers exact test (two-sided).

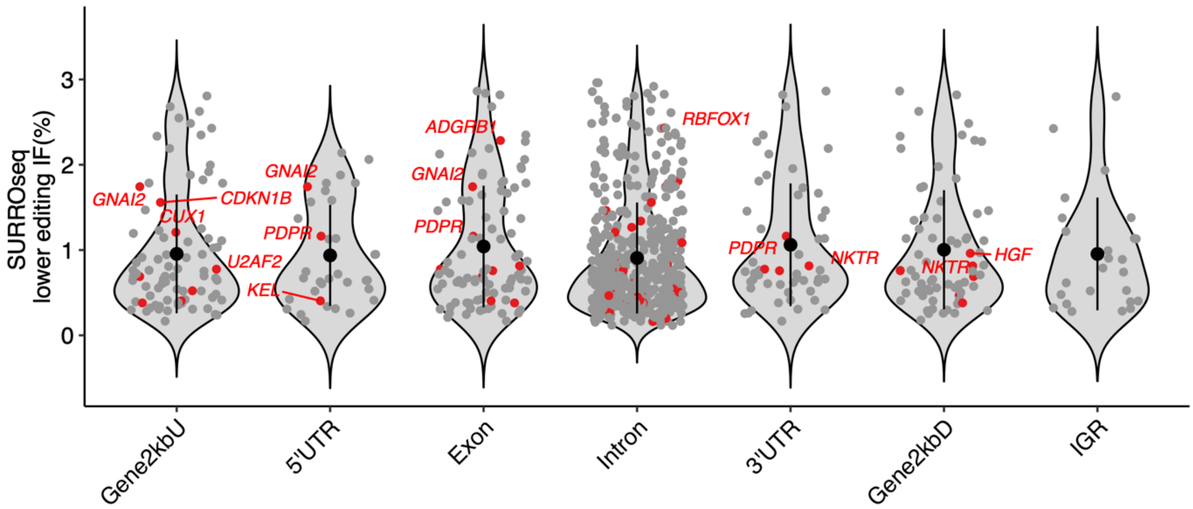

Supplementary Figure S9. Gene and genomic distribution of off-target sites with significantly detectable indel but with indel frequency below 3%. Violin plot of indel frequency, line value is mean and 1sd. OTs located in cancer genes are highlighted in red. IGR, intergenic region. UTR, untranslated region.

Supplementary Figure S10 Dotplot of inde frequencies in SpCas9 and MOCK (page 1/10)

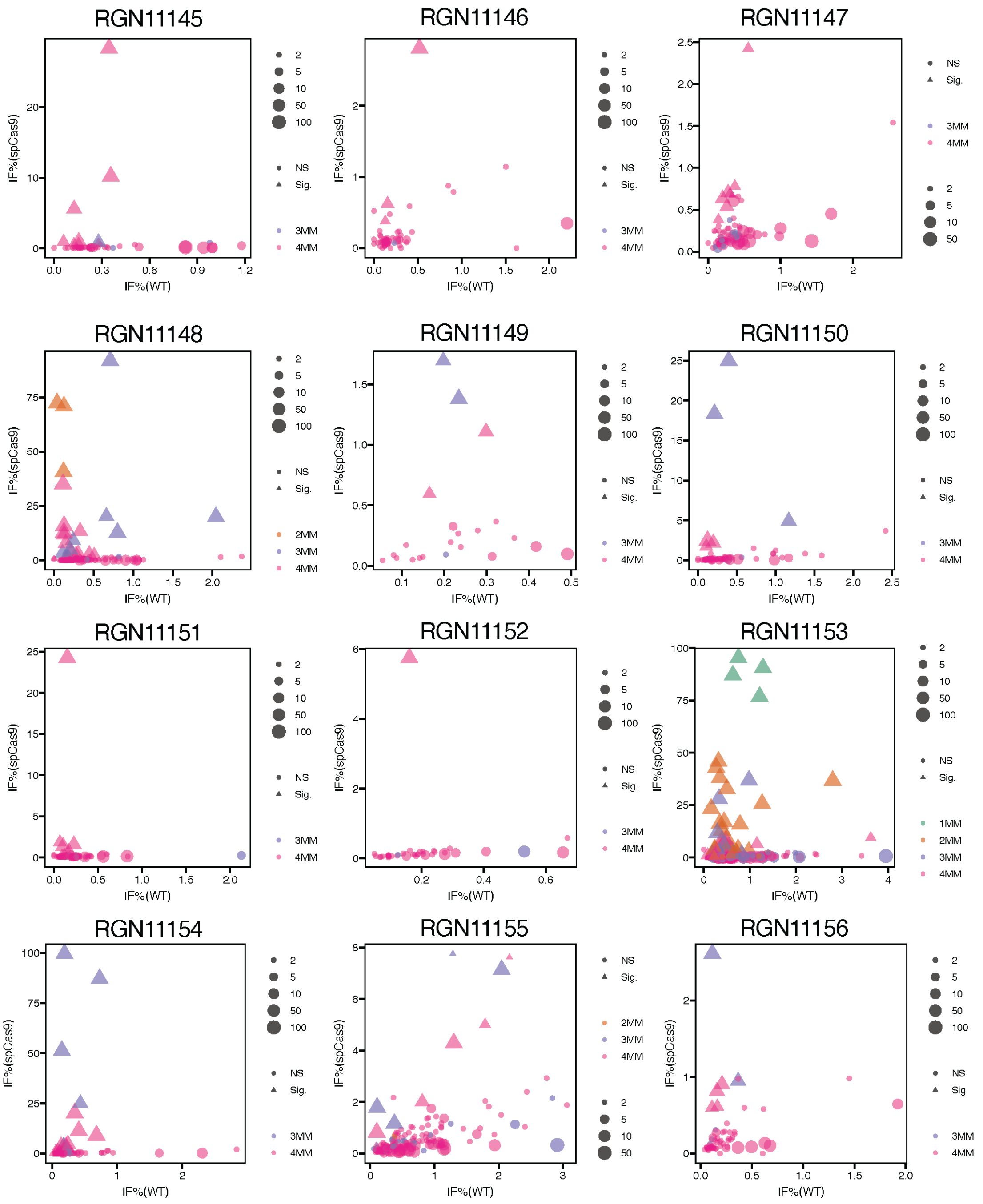

Supplementary Figure S10 (page 2/10)

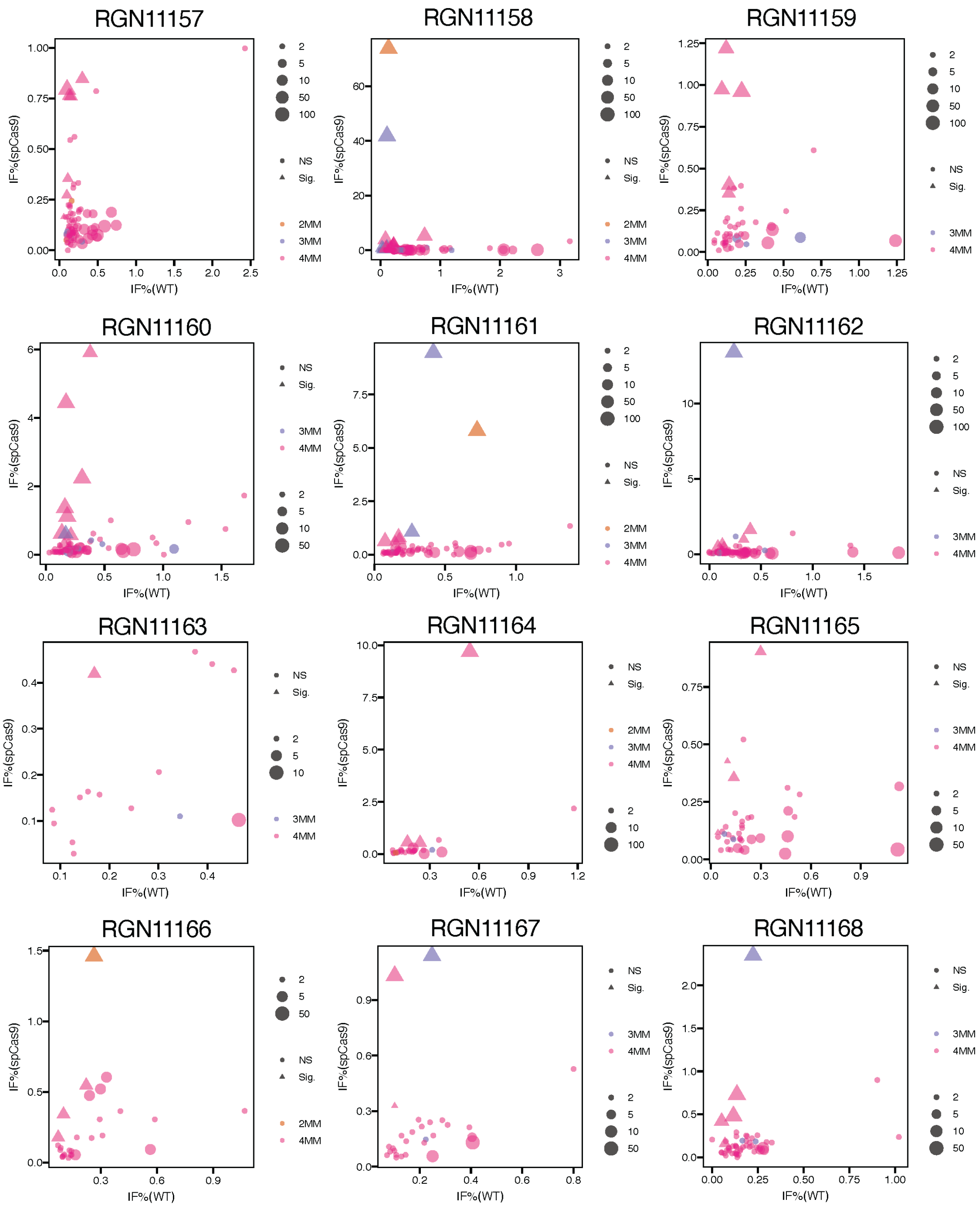

Supplementary Figure S10 (page 3/10)

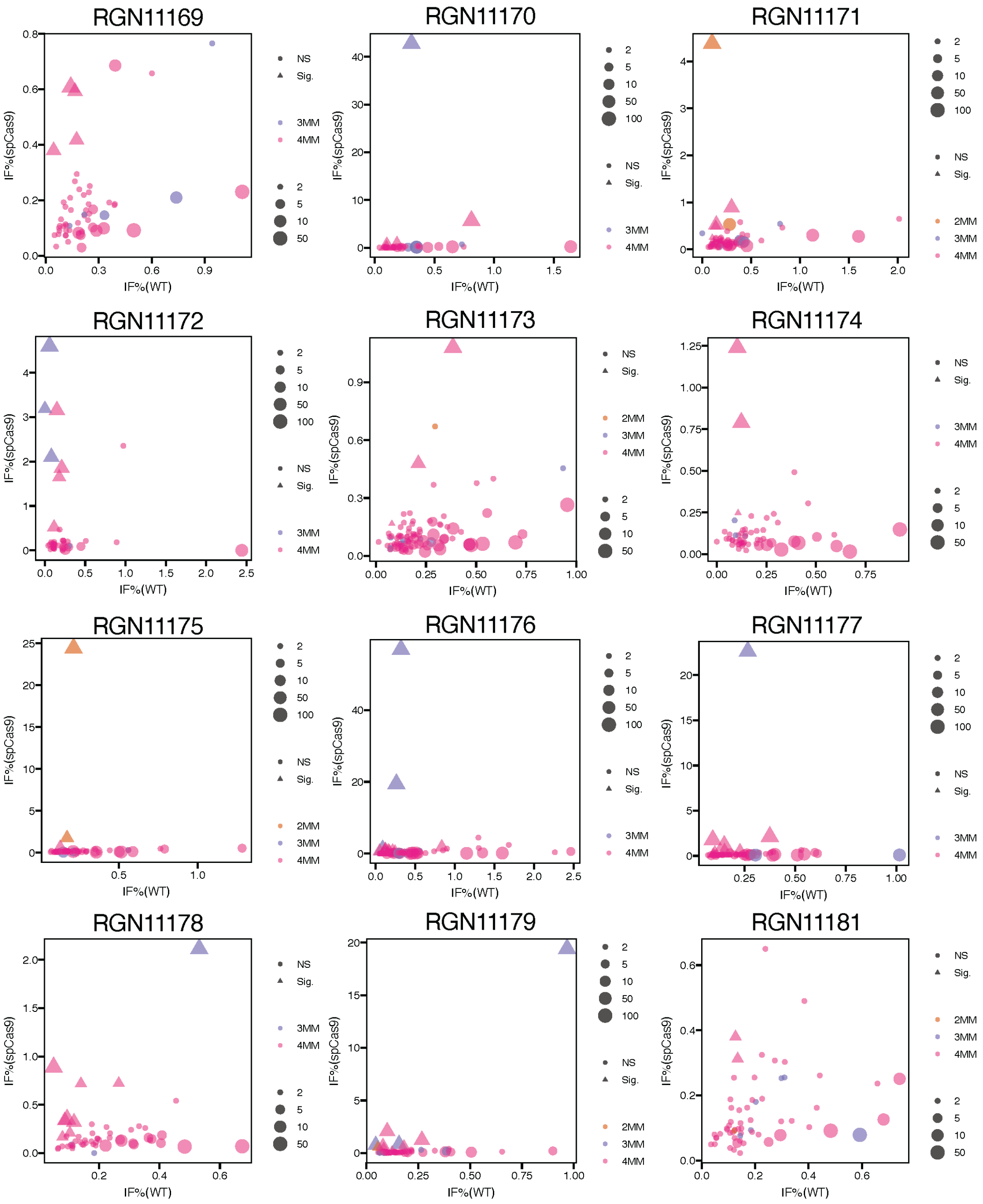

Supplementary Figure S10 (page 4/10)

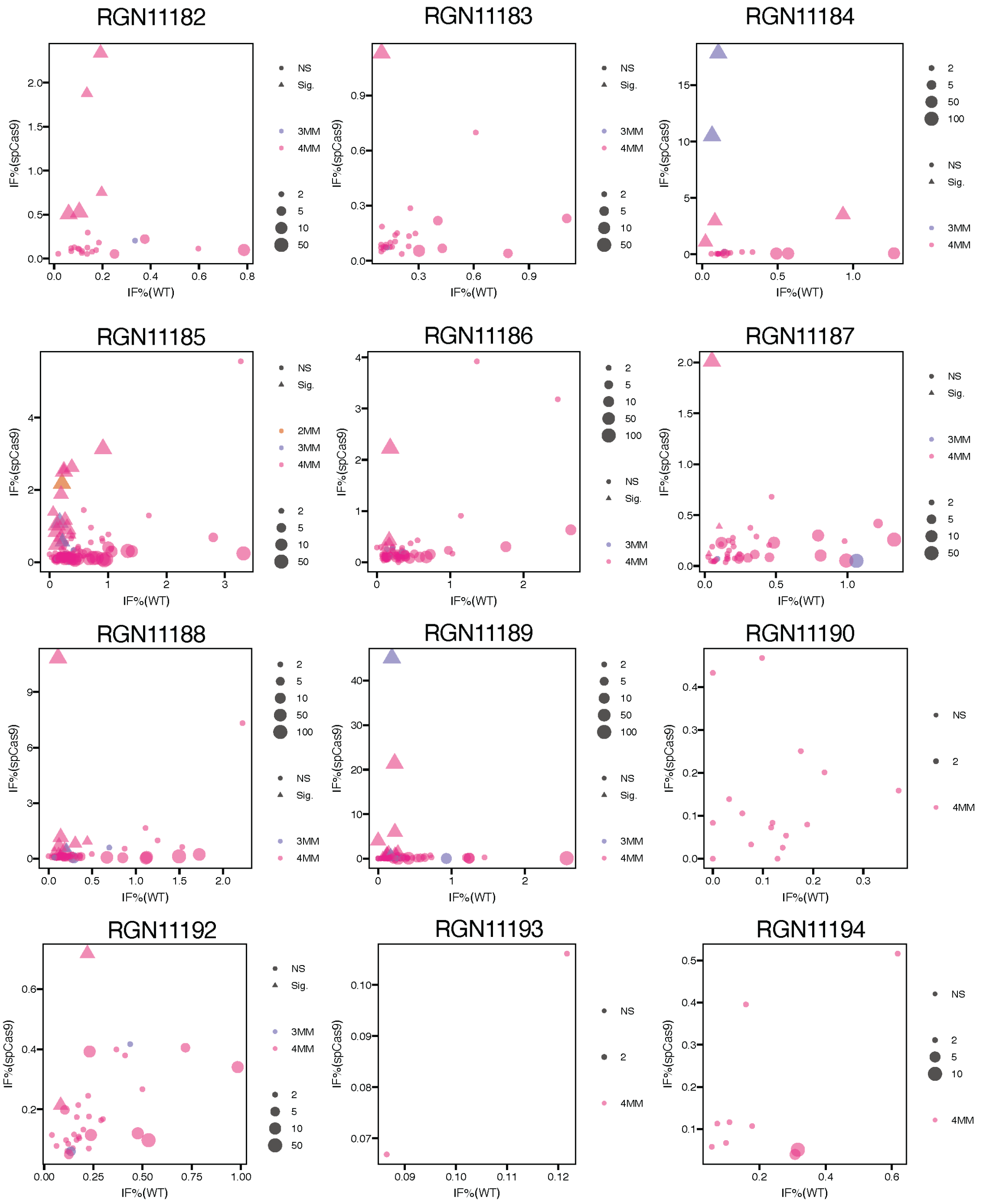

Supplementary Figure S10 (page 5/10)

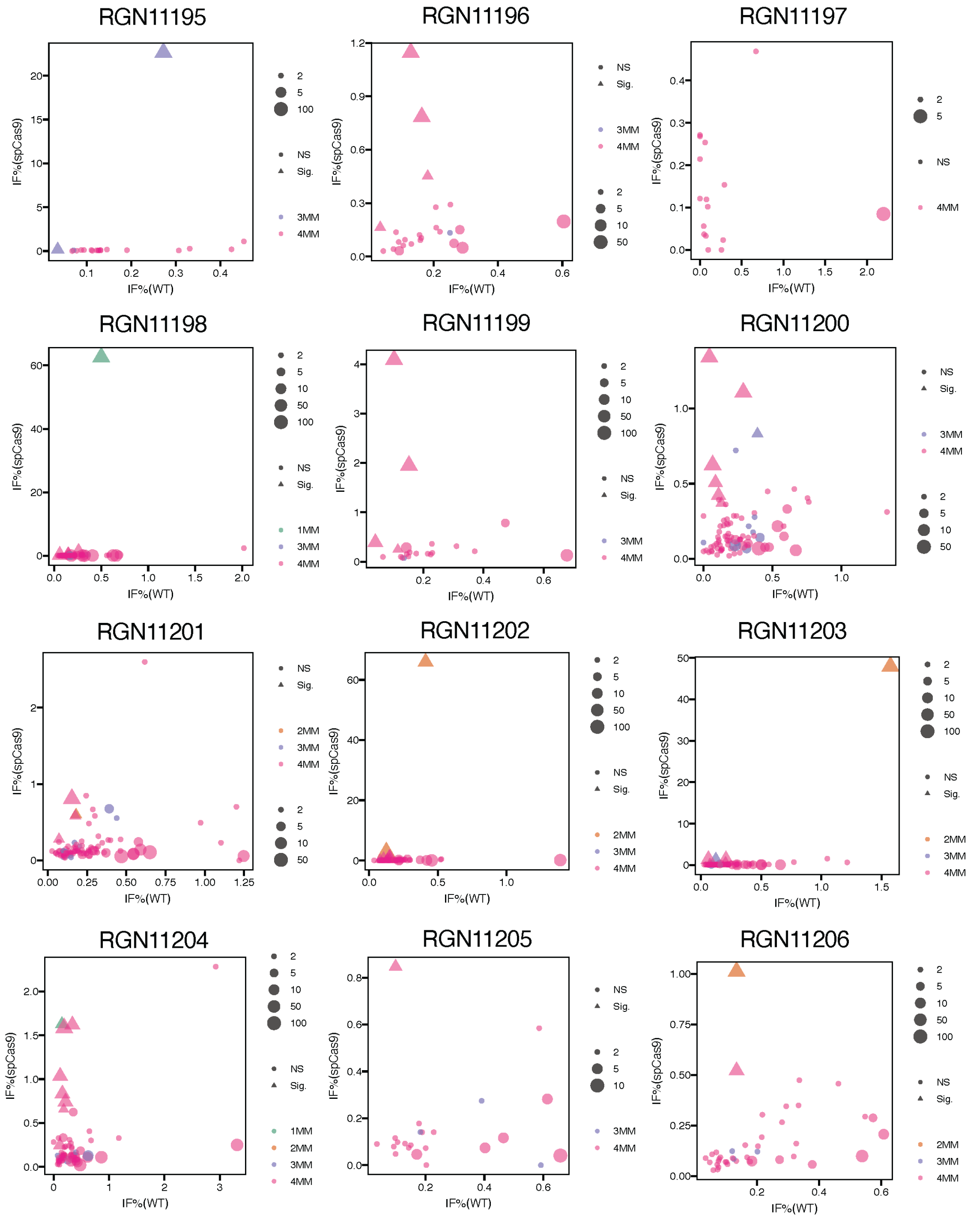

Supplementary Figure S10 (page 6/10)

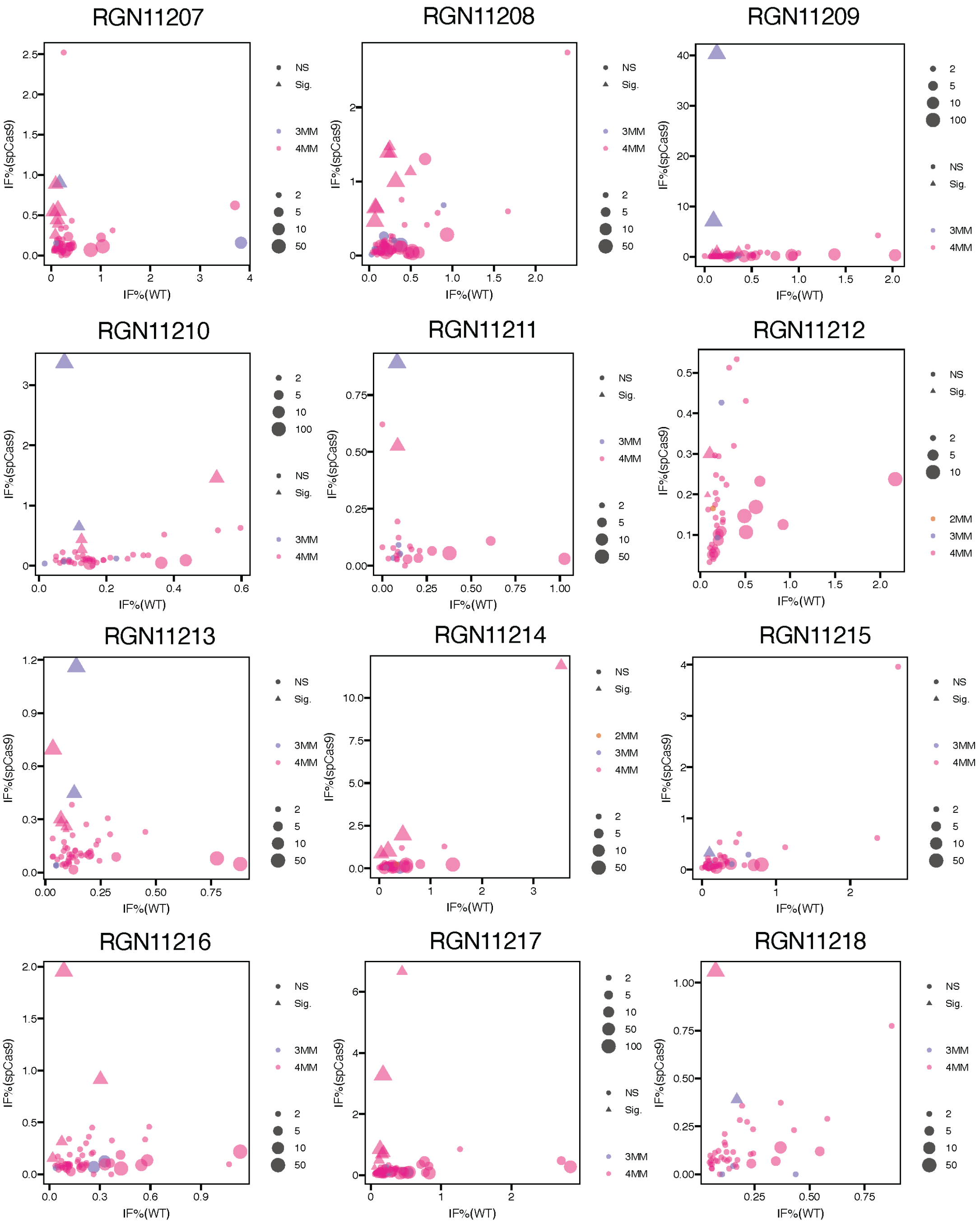

Supplementary Figure S10 (page 7/10)

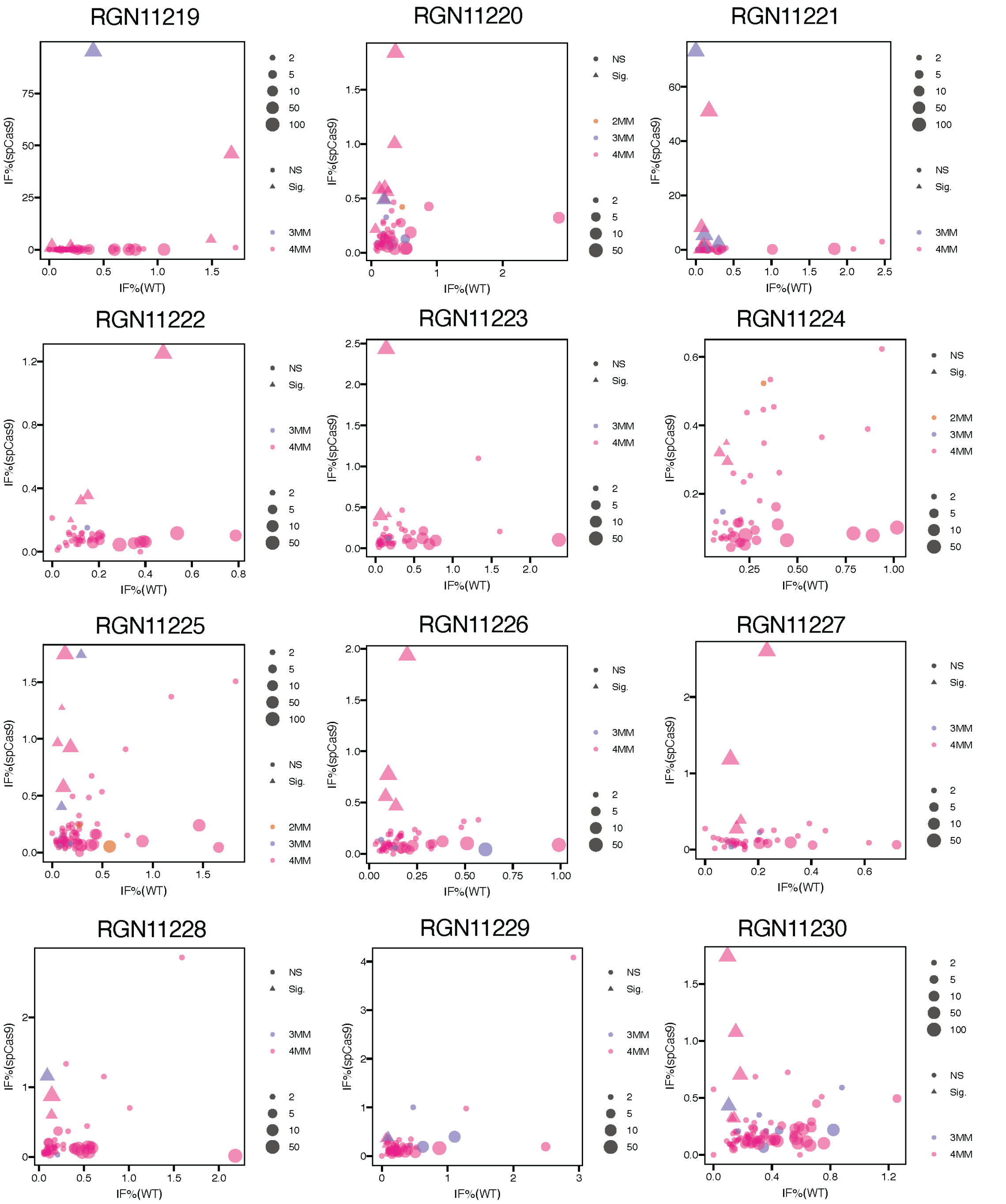

Supplementary Figure S10 (page 8/10)

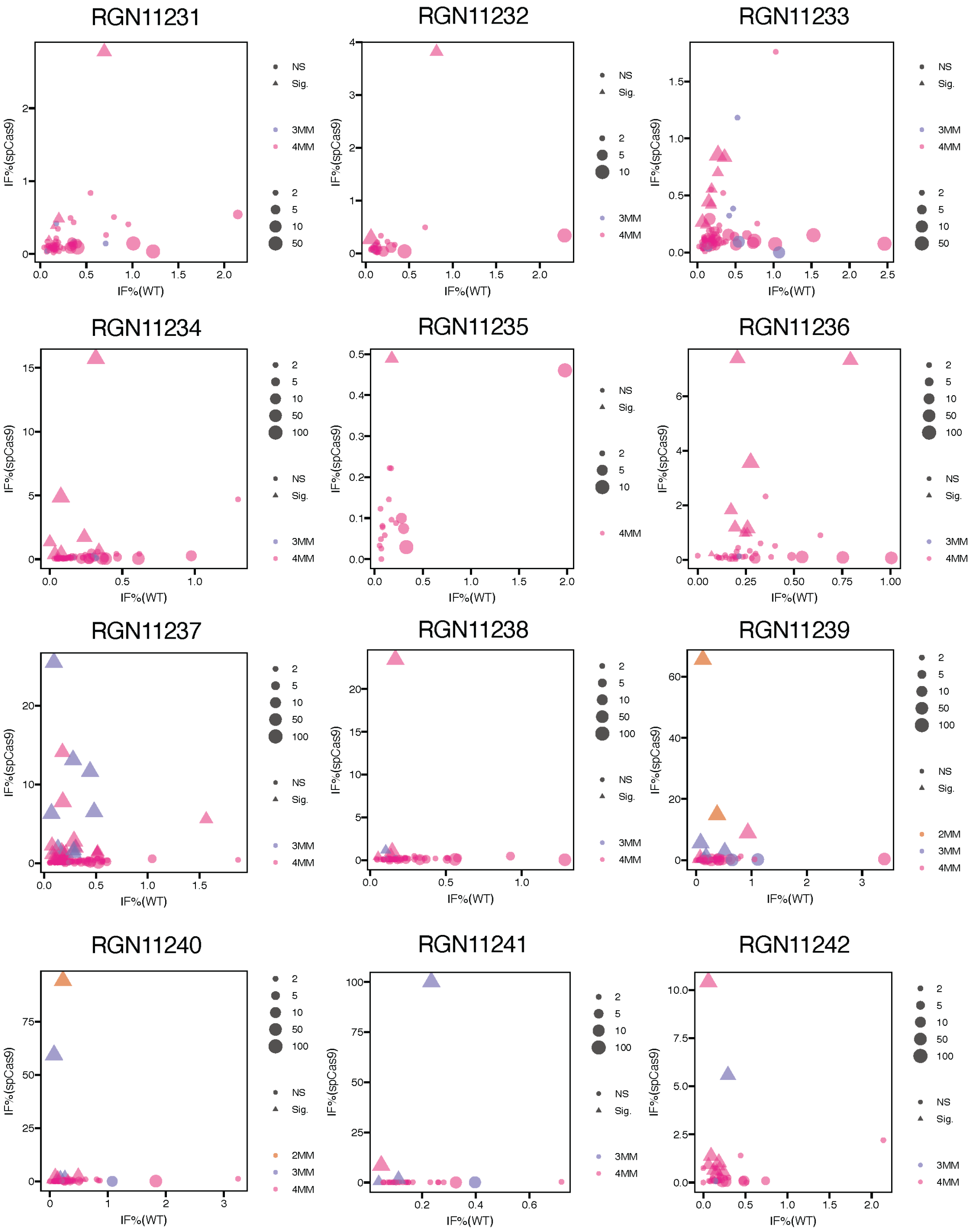

Supplementary Figure S10 (page 9/10)

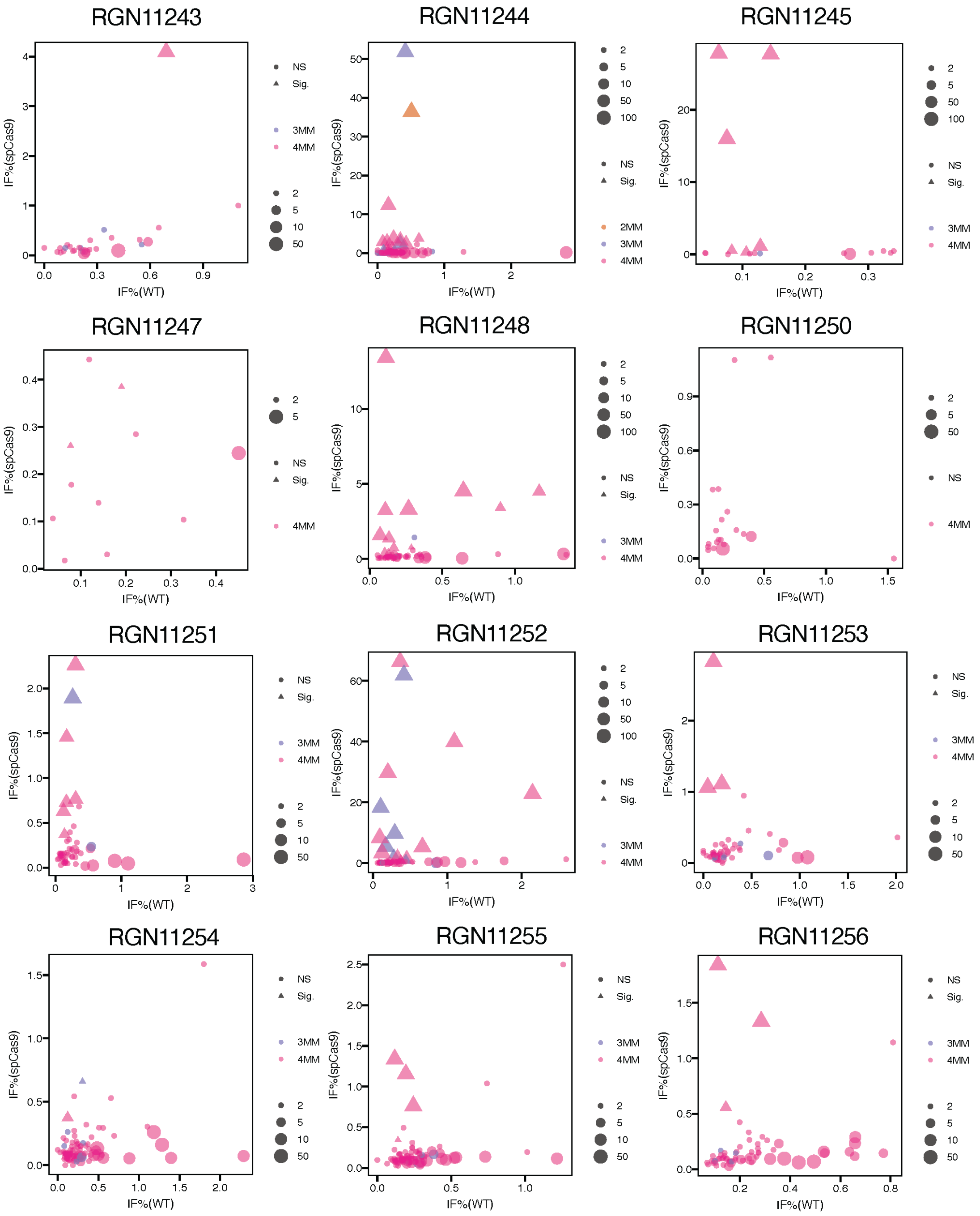

Supplementary Figure S10 (page 10/10)

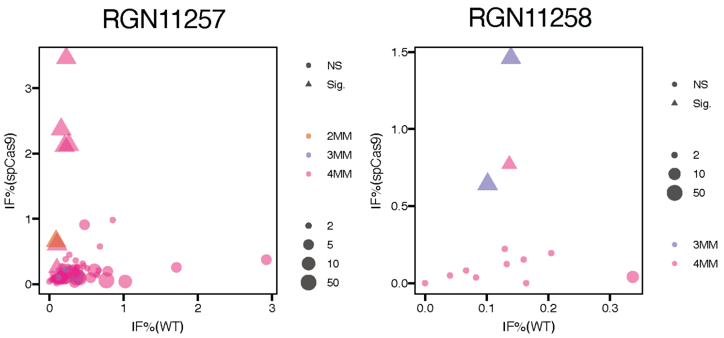

Supplementary Figure S10. Dot plot of indel frequencies for all OTs measured in each RGN by SURRO-seq. For each RGN, the indel frequency of all OTs measured SURRO-seq was plotted between MOCK and SpCas9. Each OT dot is stratified according to -log10 (adj.p value, Benjamini and Hochberg (BH)-adjusted Fisher's exact test (two-sided)). NS, not significant; MM, number of mismatches between OT and the RGN on-target.

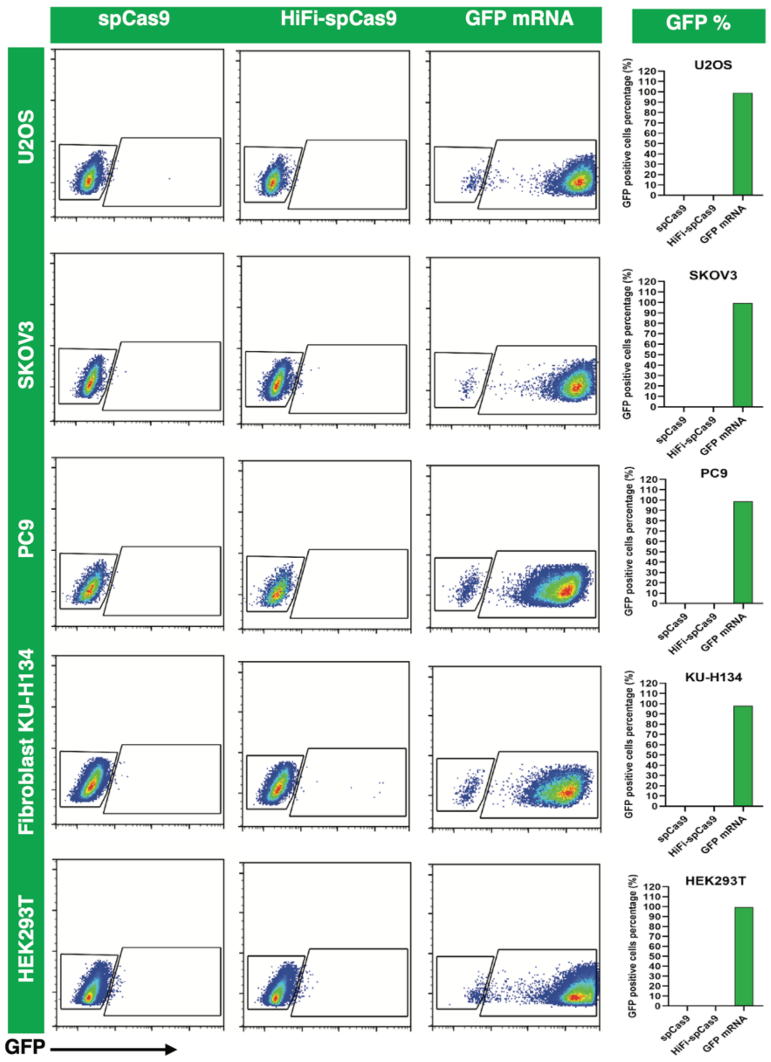

Supplementary Figure S11. Quantification of nucleofection efficiency with GFP mRNA in five human cell lines. Five human cell lines (U2OS, SKOV-3, PC9, Fibroblasts, HEK293T) are nucleofected with SpCas9 protein, HiFi-SpCas9 protein, or GFP mRNA. 48 hours after transfection, GFP positive cells is measured by flow cytometry. Right, bar plot of %GFP positive cells. Replicate = 1. Number of cells analyzed > 10,000.

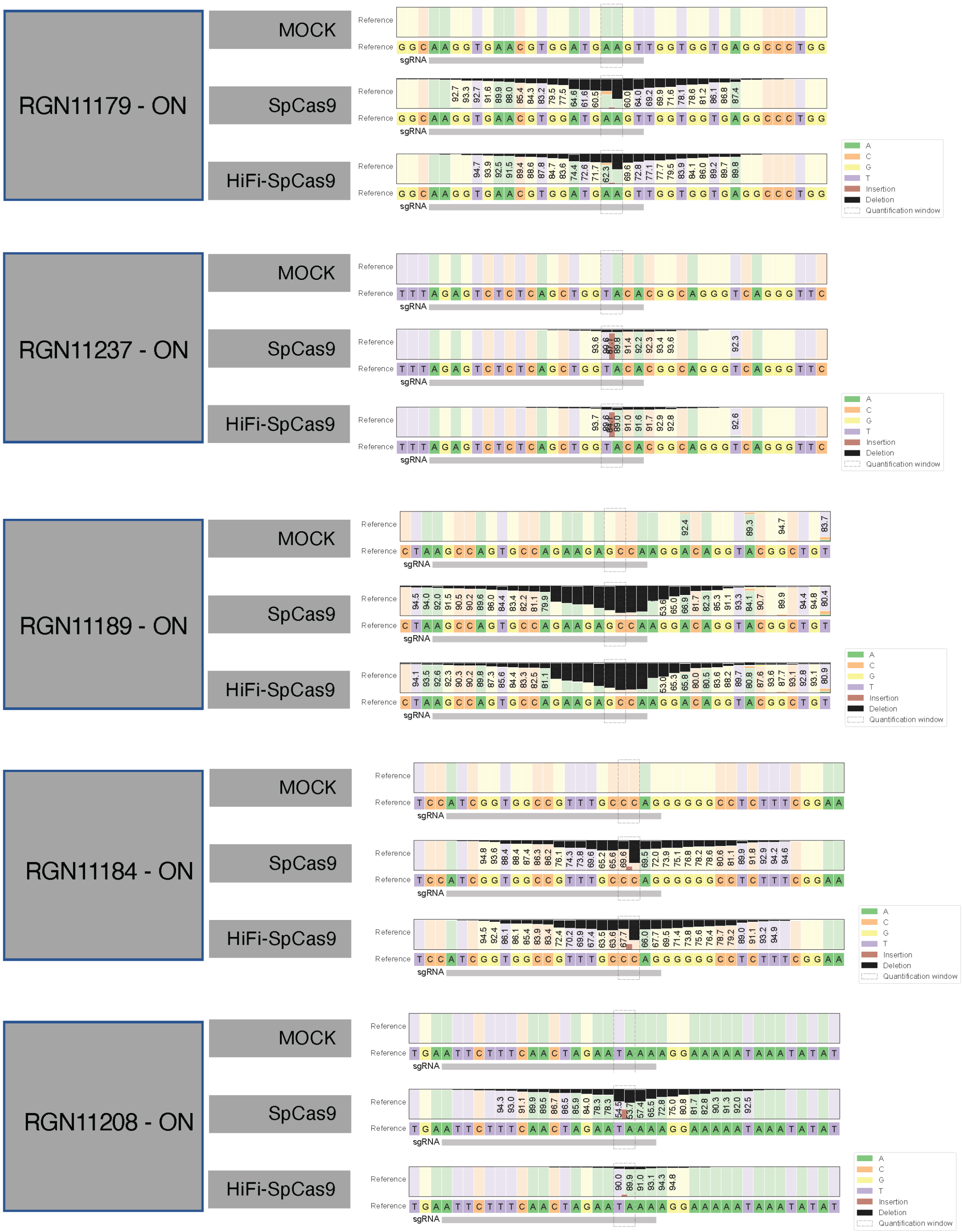

RGN11179 - ON
RGN11237 - ON
RGN11189 - ON
RGN11184 - ON
RGN11208 - ON
MOCK
SpCas9
HiFi-SpCas9
Reference
sgRNA
A
C
G
T
Insertion
Deletion
Quantification window

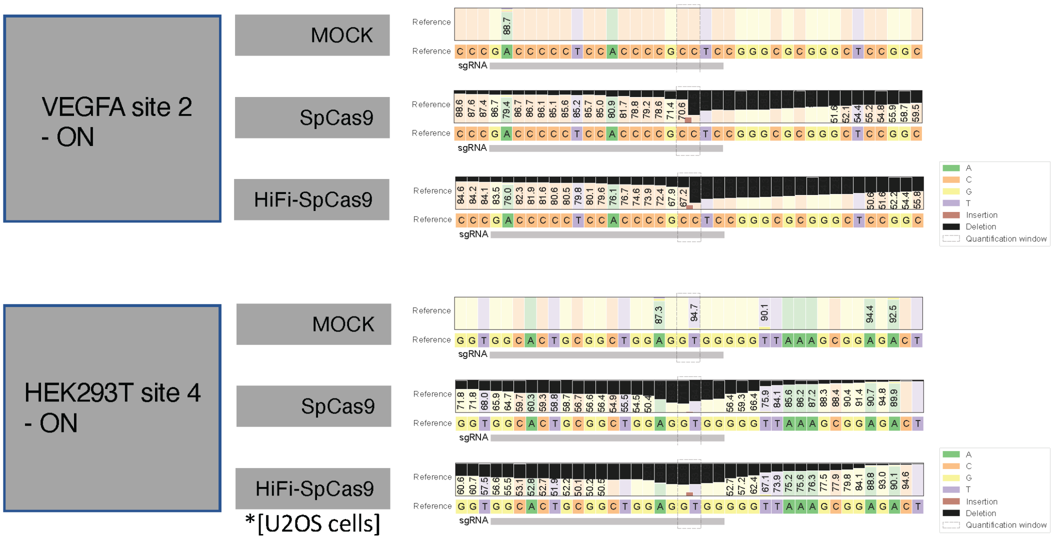

Supplementary Figure S12. Deep sequencing analysis of 7 RGN-edited on-target sites in HEK293T cells. All deep sequencing data can be found in the CNGB data depository and summarized in Supplementary Data 5 and 7. Figures presented are nucleotide percentage plots and Deep sequencing data is analyzed with CRISPResso2. Note. Data for HEK293T site 4 edited with HiFi-SpCas9 is from U2OS cells. Other data showed here are from HEK293T cells.

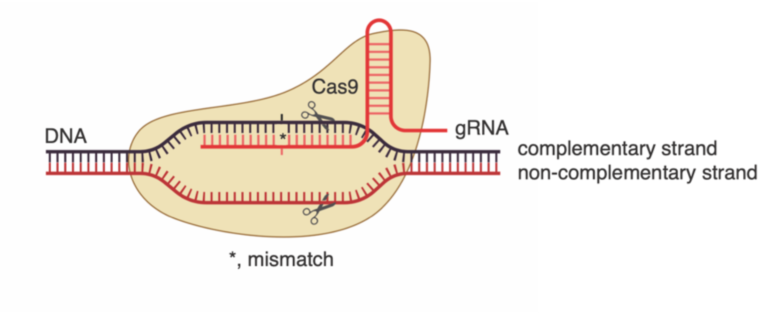

Mismatch Types

on-target protospacer: off-target protospacer		gRNA spacer:complementary strand
A:T	→	rA:dA
A:C	→	rA:dG
A:G	→	rA:dC
T:A	→	rU:dT
T:C	→	rU:dG
T:G	→	rU:dC
C:A	→	rC:dT
C:T	→	rC:dA
C:G	→	rC:dC
G:A	→	rG:dT
G:T	→	rG:dA
G:C	→	rG:dG

Supplementary Figure S13. Graphical illustration of mismatch types. Top, schematic drawing of SpCas9 in complex with gRNA and the off-target with one mismatch. Drawn with www.Biorender.com (with license for publication). Bottom (left), corresponding mismatch type between the on-target protospacer and the off-target protospacer (off-target site). Bottom (right), mismatch type between gRNA and the complementary strand (targeting strand).

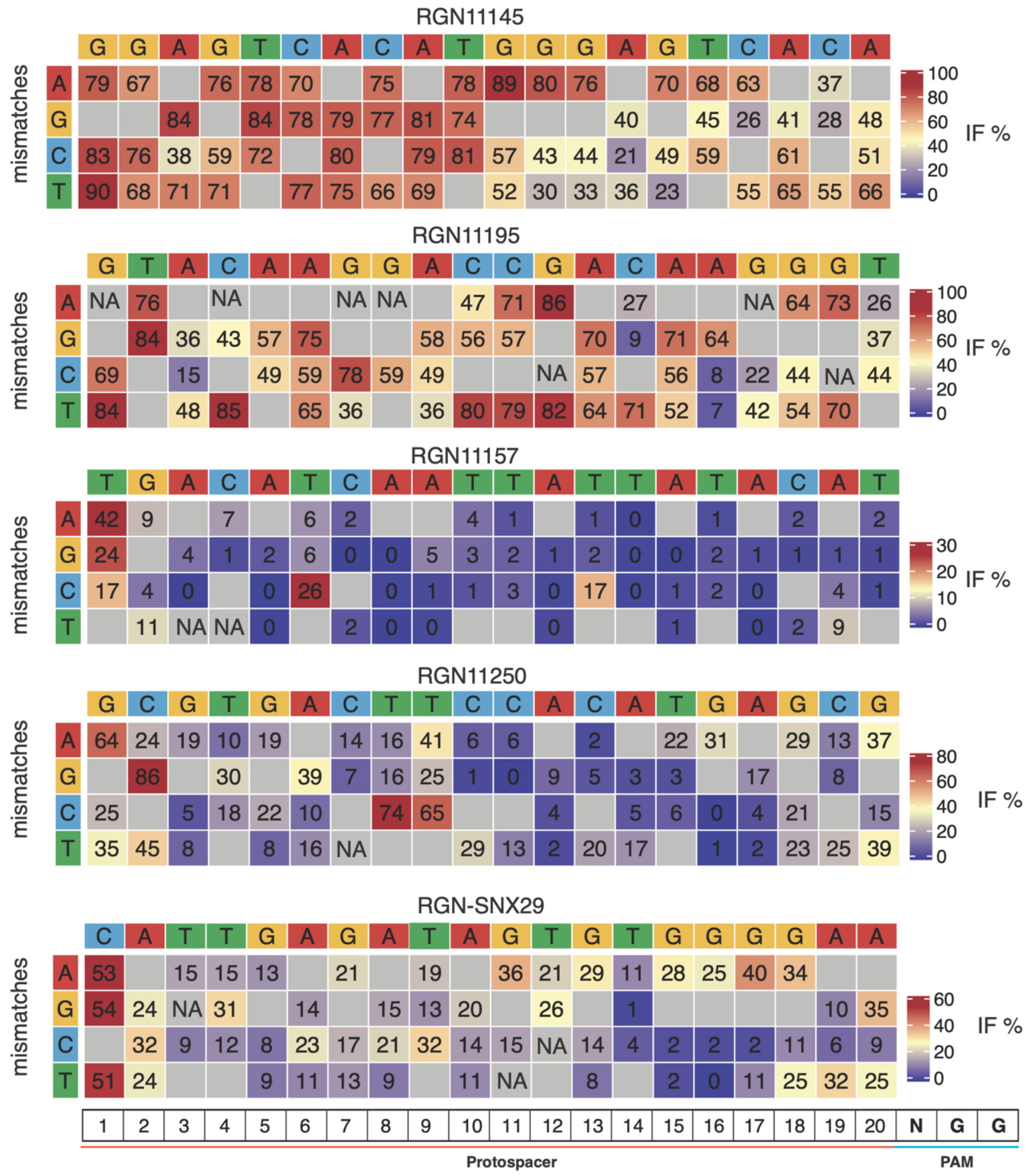

Supplementary Figure S14. Systematic quantification of off-target sites with one mismatch.

Heatmap plot of indel frequency for off-target site with one mismatch at each position and each mismatch type. NA, value not available (drop out in sequencing). IF, indel frequency. PAM, protospacer adjacent motif.

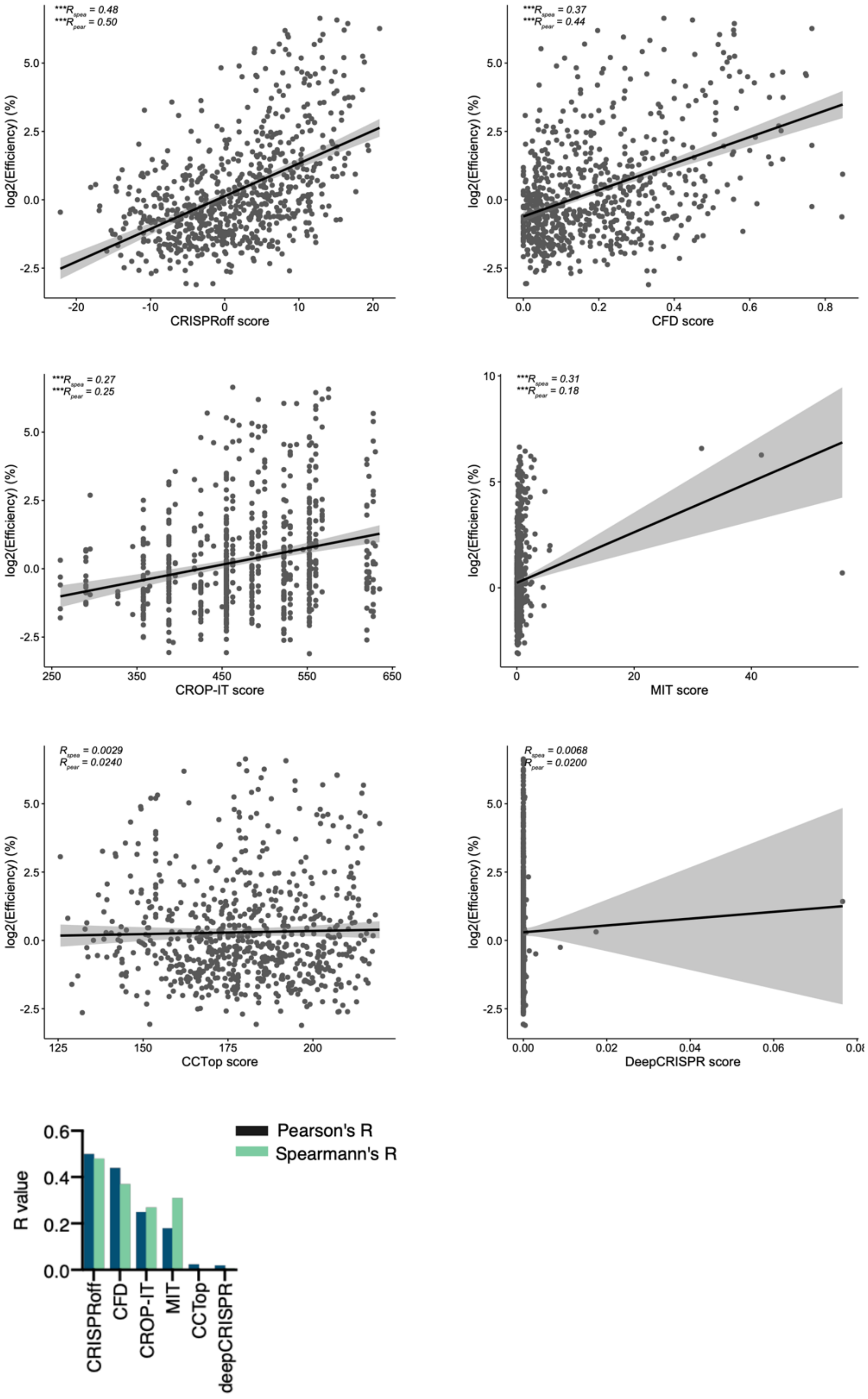

Supplementary Figure S15. Benchmark analysis of six RGN off-target prediction method with LibB sig. OTs. Pearson's and Spearman' correlations were analyzed between the log2 indel frequency measured by SURRO-seq and the corresponding off-target score predicted by each method. Data are presented as fitted lines (Linear Regression) and 95% confidence intervals (shadow). Bottom, summary of Pearson and Spearman's R values in bar plot.

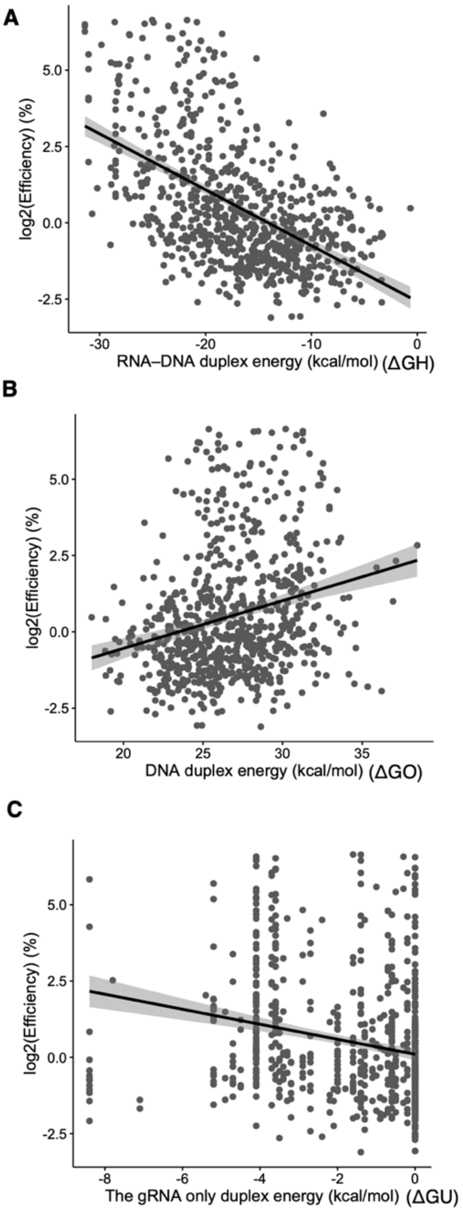

Supplementary Figure S16. Correlation between indel frequency and three energy features.

A. Correlation between indel frequency of the Sig. OTs captured by SURRO-seq and the gRNA-DNA duplex energy.

B. Correlation between indel frequency of the Sig. OTs captured by SURRO-seq and the DNA duplex opening energy.

C. Correlation between indel frequency of the Sig. OTs captured by SURRO-seq and the gRNA only duplex energy. The gRNA only duplex energy was calculated for the on-target RGN gRNA spacer only.

Data are presented as fitted lines (Linear Regression) and 95% confidence intervals (shadow).

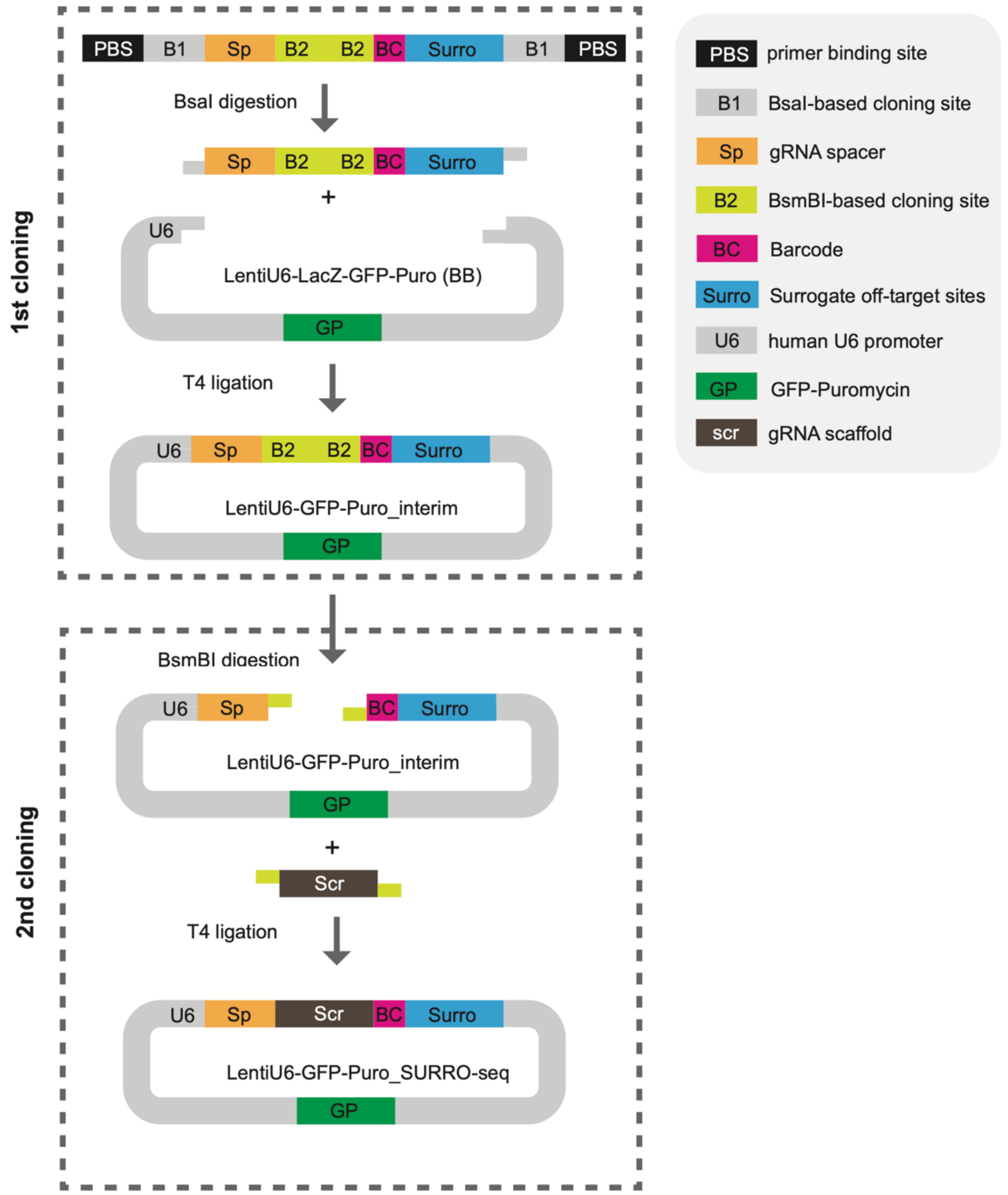

Supplementary Figure S17. A proposed two-round cloning strategy to generate the SURRO-seq library. This aim of this proposed two-round cloning strategy is to overcome the length limitation of synthesizing the SURRO-seq oligonucleotide pool, as well as reducing the synthetic errors introduced into the gRNA scaffold.

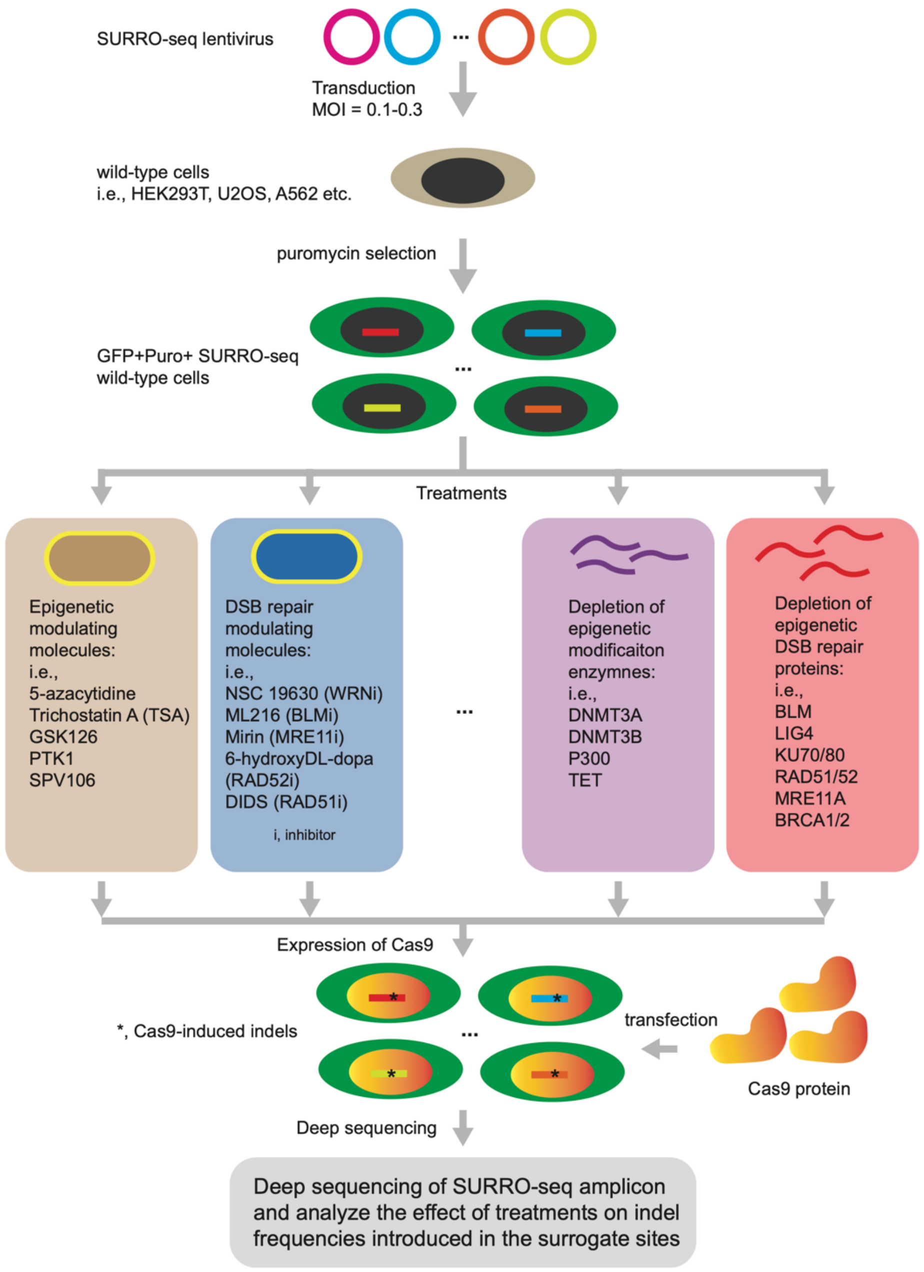

Supplementary Figure S18. Potential application of SURRO-seq for studying the effect of gRNA sequences-independent factors on RGN activity and specificity.

The SURRO-seq libraries, e.g., on-target library generated by us previously[4] and off-target library generated in this study, are stably integrated into wildtype modelling cells. These SURRO-seq carrying cells will then subjected to treatments of interests. For example, we illustrated here with small chemical molecule treatments (left panels) or with targeted genetic modulations (siRNA, right panels) targeting the epigenetic modification pathways, DNA double-strand break (DSB) repair pathways. Expression of the Cas9 protein will then be introduced into the SURRO-seq cells with or without treatments, followed by quantification and evaluation of indels (*) introduced in the cells by deep sequencing and data analysis.

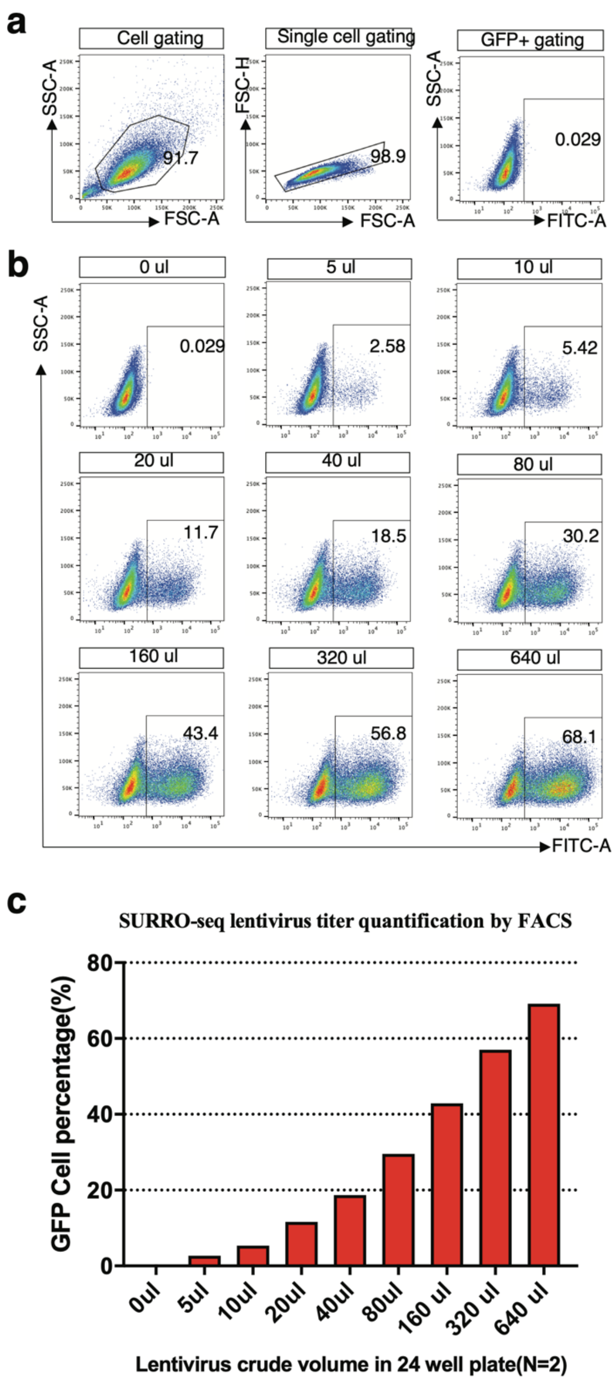

Supplementary Figure S19. Gating strategy for quantification of SURRO-seq lentivirus titer using flow cytometry. A. Representative gating for total cells, single cells, and GFP+ and GFP- cells. B. Representative scatter plots for cells transduced with different amount of SURRO-seq crude virus. Values are the % of GFP+ cells from one replicate. C. Bar plot of the GFP+ cells from two replicates.

PART 3. SUPPLEMENTARY REFERENCE

1. Haeussler, M. et al. Evaluation of off-target and on-target scoring algorithms and integration into the guide RNA selection tool CRISPOR. *Genome Biol* **17**, 148 (2016).
2. Alkan, F., Wenzel, A., Anthon, C., Havgaard, J.H. & Gorodkin, J. CRISPR-Cas9 off-targeting assessment with nucleic acid duplex energy parameters. *Genome Biol* **19**, 177 (2018).
3. Bae, S., Park, J. & Kim, J.S. Cas-OFFinder: a fast and versatile algorithm that searches for potential off-target sites of Cas9 RNA-guided endonucleases. *Bioinformatics* **30**, 1473-1475 (2014).
4. Xiang, X. et al. Enhancing CRISPR-Cas9 gRNA efficiency prediction by data integration and deep learning. *Nat Commun* **12**, 3238 (2021).
5. Laehnemann, D., Borkhardt, A. & McHardy, A.C. Denoising DNA deep sequencing data-high-throughput sequencing errors and their correction. *Brief Bioinform* **17**, 154-179 (2016).
