# Supplementary material for: Massively targeted evaluation of therapeutic CRISPR off-targets in cells
Source: Nat Commun. 2022 Jul 13;13:4049. doi: 10.1038/s41467-022-31543-6 (PMC9279339; doi:10.1038/s41467-022-31543-6)
Supplement: Supplementary file 2 — Reporting Summary [file 41467_2022_31543_MOESM2_ESM.pdf]

nature research

Corresponding author(s): George M. Church, Jan Gorodkin, Lin Lin, Yonglun Luo

Last updated by author(s): Jun 3, 2022

Reporting Summary

Nature Research wishes to improve the reproducibility of the work that we publish. This form provides structure for consistency and transparency in reporting. For further information on Nature Research policies, see our Editorial Policies and the Editorial Policy Checklist.

Statistics

For all statistical analyses, confirm that the following items are present in the figure legend, table legend, main text, or Methods section.

n/a	Confirmed	
☐	☒	The exact sample size (*n*) for each experimental group/condition, given as a discrete number and unit of measurement
☐	☒	A statement on whether measurements were taken from distinct samples or whether the same sample was measured repeatedly
☐	☒	The statistical test(s) used AND whether they are one- or two-sided<br>*Only common tests should be described solely by name; describe more complex techniques in the Methods section.*
☒	☐	A description of all covariates tested
☒	☐	A description of any assumptions or corrections, such as tests of normality and adjustment for multiple comparisons
☐	☒	A full description of the statistical parameters including central tendency (e.g. means) or other basic estimates (e.g. regression coefficient) AND variation (e.g. standard deviation) or associated estimates of uncertainty (e.g. confidence intervals)
☐	☒	For null hypothesis testing, the test statistic (e.g. *F*, *t*, *r*) with confidence intervals, effect sizes, degrees of freedom and *P* value noted<br>*Give P values as exact values whenever suitable.*
☒	☐	For Bayesian analysis, information on the choice of priors and Markov chain Monte Carlo settings
☒	☐	For hierarchical and complex designs, identification of the appropriate level for tests and full reporting of outcomes
☒	☐	Estimates of effect sizes (e.g. Cohen's *d*, Pearson's *r*), indicating how they were calculated

Our web collection on statistics for biologists contains articles on many of the points above.

Software and code

Policy information about availability of computer code

Data collection	no software was used
Data analysis	Off-target sites of each RGN gRNA were searched with FlashFry (v 1.80). The FCM output data was analyzed by the software Flowjo vX.0.7. For raw data processing, FastaQC-v0.11.3(https://www.bioinformatics.babraham.ac.uk/projects/fastqc/) and fastp-v0.19.6(https://github.com/OpenGene/fastp) with default options were used for data quality control and filtering with the default parameters. The pair-end data was assembled using FLASH-v1.2.11 (http://www.cbcb.umd.edu/software/flash). BWA-MEM-v0.7.17 with default options was used to map the assembled data to the designed oligos sequence to preliminarily distinguish the data of each surrogate site. The pysam module of Python-3.8 was used to split the aligned data according to the site number of the chip. The julia-1.5.3 language was used for data filtering. Fisher's exact test (two-sided, adjusted by BH) and other statistical analysis were performed in R-4.0.3.

For manuscripts utilizing custom algorithms or software that are central to the research but not yet described in published literature, software must be made available to editors and reviewers. We strongly encourage code deposition in a community repository (e.g. GitHub). See the Nature Research guidelines for submitting code & software for further information.

Data

Policy information about availability of data

All manuscripts must include a data availability statement. This statement should provide the following information, where applicable:

- Accession codes, unique identifiers, or web links for publicly available datasets
- A list of figures that have associated raw data
- A description of any restrictions on data availability

The human genome hg19 was used for RGN OT searching. All NGS data generated by this study have been shared via the CNGB public data depository with the

following accession numbers: CNP0001979 [https://db.cngb.org/search/project/CNP0001979/] and CNP0002648 [https://db.cngb.org/search/project/CNP0002648/]. A complete list of 704 NGS samples were summarized in Supplementary Data 7. All other relevant data supporting the key findings of this study are available within the article and its Supplementary Information files or from the corresponding author upon reasonable request. Source data are provided with this paper.

Field-specific reporting

Please select the one below that is the best fit for your research. If you are not sure, read the appropriate sections before making your selection.

- [x] Life sciences
- [ ] Behavioural & social sciences
- [ ] Ecological, evolutionary & environmental sciences

For a reference copy of the document with all sections, see nature.com/documents/nr-reporting-summary-flat.pdf

Life sciences study design

All studies must disclose on these points even when the disclosure is negative.

Sample size	No sample size calculation was performed. To validate the SURRO-seq method and its broad applicability, we have designed three libraries (LibA, LibB, and LibC). LibA contains 11 RGNs and 170 OTs. LibB contains 110 RGNs and 8150 OTs. And LibC contrains 5 RGNs and 300 OTs. For the validation of SURRO-seq captured OTs, we have evaluated 23 OTs for 7 RGNs in five human cell lines. The large sample sizes of both RGNs and OTs are sufficient to support the broad applicability and validity of SURRO-seq.
Data exclusions	To exclusion reads with low synthetic quality during the oligonucleotide synthesis and sites which might affect cell growth, this study used the following filtering steps to exclude low quality sites. (1) Total clean reads less than 32 for both MOCK and SpCas9. (2) percentage indel frequency in MOCK cells above 4%. (3) Fold change of enrichment or depletion between SpCas9 edited and MOCK cells larger than 2 folds.
Replication	The SURRO-seq method has been validated with three libraries. All attempts at replication were successful.
Randomization	In this study, the experimental work and the computational (including the design of SURRO-seq libraries and the analysis of SURRO-seq captured indels) were performed by two groups of investigators. When synthesizing the SURRO-seq oligonucleotides, we randomized the order of the synthetic RGNs oligonucleotides in the chip. For the SURRO-seq data analysis, it is not possible to randomize the data as we have to analyze the indels for each RGN and surrogate off-targets.
Blinding	For the experimental parts of the study, the investigators were not blinded to the group of transduction as their have to know which cell lines and their culture conditions. For the sequencing and NGS data collection, the NGS team was blinded to the sequencing libraries. All the NGS libraries were labeled with numeric ID without information from MOCK or Cas9. For the data analysis, it is not possible to apply blinding as the bioinformatic team need the information of experiment groups and the SURRO-seq reference sequences in order to calculate the indels.

Reporting for specific materials, systems and methods

We require information from authors about some types of materials, experimental systems and methods used in many studies. Here, indicate whether each material, system or method listed is relevant to your study. If you are not sure if a list item applies to your research, read the appropriate section before selecting a response.

Materials & experimental systems

n/a	Involved in the study	
[x]	[ ]	Antibodies
[ ]	[x]	Eukaryotic cell lines
[x]	[ ]	Palaeontology and archaeology
[x]	[ ]	Animals and other organisms
[x]	[ ]	Human research participants
[x]	[ ]	Clinical data
[x]	[ ]	Dual use research of concern

Methods

n/a	Involved in the study	
[x]	[ ]	ChIP-seq
[ ]	[x]	Flow cytometry
[x]	[ ]	MRI-based neuroimaging

Eukaryotic cell lines

Policy information about cell lines

Cell line source(s)	SpCas9-expressing HEK293T cells were generated by the Luo lab previously. The wild type HEK293T, U2OS, and SKOV-3 were from ATCC. The PC9 cells were purchased from Merck. Human primary fibroblasts were established by the Luo lab.
Authentication	All the cell lines used in this study are commercially available and authenticated by the original sources. Primary fibroblasts are established from previous studies from the Luo group.
Mycoplasma contamination	All cells were tested negative for mycoplasma contamination.
Commonly misidentified lines (See ICLAC register)	There is no commonly misidentified cell lines used by the study.

Flow Cytometry

Plots

Confirm that:

- [x] The axis labels state the marker and fluorochrome used (e.g. CD4-FITC).
- [x] The axis scales are clearly visible. Include numbers along axes only for bottom left plot of group (a 'group' is an analysis of identical markers).
- [x] All plots are contour plots with outliers or pseudocolor plots.
- [x] A numerical value for number of cells or percentage (with statistics) is provided.

Methodology

Sample preparation	Cultured cells were trypsinized and resuspended in PBS. Cell suspensions were analyzed with a NovoCyte Quanteon flow cytometer with 4 lasers.
Instrument	NovoCyte Quanteon flow cytometer with 4 lasers provided by the FACS CORE facility at the Department of Biomedicine, Aarhus University.
Software	Data collection and analysis were performed using the software included in the NovoCyte Quanteon flow cytometer.
Cell population abundance	Flow cytometry was used to quantify the fraction of GFP positive cells. In our experiment, the GFP positive cells are the major population in cells transfected with an EGFP mRNA.
Gating strategy	The major cell population was gated based FSC-A/SSC-A to select the major cell populated. Singlets were gated based on FSC-A and FSC-H. Un-transfected cells were used for setting the gate for GFP negative cells. At least 10,000 cells were collected for each sample.

- [x] Tick this box to confirm that a figure exemplifying the gating strategy is provided in the Supplementary Information.
