# Supplementary material for: Massively targeted evaluation of therapeutic CRISPR off-targets in cells
Source: Nat Commun. 2022 Jul 13;13:4049. doi: 10.1038/s41467-022-31543-6 (PMC9279339; doi:10.1038/s41467-022-31543-6)
Supplement: Supplementary file 3 — Description of Additional Supplementary Files [file 41467_2022_31543_MOESM3_ESM.pdf]

Title: Supplementary Data 1.
Description: A noncomprehensive list of RGN offtarget detection methods.

Title: Supplementary Data 2.
Description: SURRO-seq LibA data.

Title: Supplementary Data 3.
Description: SURRO-seq LibB data.

Title: Supplementary Data 4.
Description: Deep sequencing of RGN-edited endogenous on-target and off-target sites in five human cell lines.

Title: Supplementary Data 5.
Description: Mismatch calculation and SURRO-seq LibC data.

Title: Supplementary Data 6.
Description: Primer sequences.

Title: Supplementary Data 7.
Description: List of NGS sequencing data depository
